# 46,XX DSD Due to Androgen Excess in Monogenic Disorders of Steroidogenesis: Genetic, Biochemical, and Clinical Features

**DOI:** 10.3390/ijms20184605

**Published:** 2019-09-17

**Authors:** Federico Baronio, Rita Ortolano, Soara Menabò, Alessandra Cassio, Lilia Baldazzi, Valeria Di Natale, Giacomo Tonti, Benedetta Vestrucci, Antonio Balsamo

**Affiliations:** 1 Pediatric Unit, Department of Medical and Surgical Sciences, S.Orsola-Malpighi University Hospital, 40138 Bologna, Italy; 2 Genetic Unit, Department of Medical and Surgical Sciences, S.Orsola-Malpighi University Hospital, 40138 Bologna, Italy

**Keywords:** 46,XX DSD, 21-hydroxylase deficiency, 11-hydroxylase deficiency, 3β-hydroxysteroid dehydrogenase deficiency, aromatase deficiency, POR deficiency, androgen excess

## Abstract

The term ‘differences of sex development’ (DSD) refers to a group of congenital conditions that are associated with atypical development of chromosomal, gonadal, or anatomical sex. Disorders of steroidogenesis comprise autosomal recessive conditions that affect adrenal and gonadal enzymes and are responsible for some conditions of 46,XX DSD where hyperandrogenism interferes with chromosomal and gonadal sex development. Congenital adrenal hyperplasias (CAHs) are disorders of steroidogenesis that mainly involve the adrenals (21-hydroxylase and 11-hydroxylase deficiencies) and sometimes the gonads (3-beta-hydroxysteroidodehydrogenase and P450-oxidoreductase); in contrast, aromatase deficiency mainly involves the steroidogenetic activity of the gonads. This review describes the main genetic, biochemical, and clinical features that apply to the abovementioned conditions. The activities of the steroidogenetic enzymes are modulated by post-translational modifications and cofactors, particularly electron-donating redox partners. The incidences of the rare forms of CAH vary with ethnicity and geography. The elucidation of the precise roles of these enzymes and cofactors has been significantly facilitated by the identification of the genetic bases of rare disorders of steroidogenesis. Understanding steroidogenesis is important to our comprehension of differences in sexual development and other processes that are related to human reproduction and fertility, particularly those that involve androgen excess as consequence of their impairment.

## 1. Introduction 

The term ‘differences of sex development’ (DSD) refers to a group of congenital conditions that are associated with atypical development of chromosomal, gonadal, or anatomical sex [1]. The most common condition that presents a normal 46,XX karyotype and DSD is congenital adrenal hyperplasia (CAH) due to severe or complete 21-hydroxylase deficiency (21-OHD; an overall incidence in the Caucasian population of ~1:30,000 female newborns) [2]. Although rarer, other monogenic defects in steroidogenesis may cause androgen excess and determine similar phenotypic conditions that are related to adrenal and/or gonadal dysfunction (Table 1) [3]. The main genetic, biochemical, and clinical/therapeutic features that apply to these rare conditions will be described in this review and are summarized in Table 2.

## 2. Synopsis of Steroidogenesis

**Figure 1:**
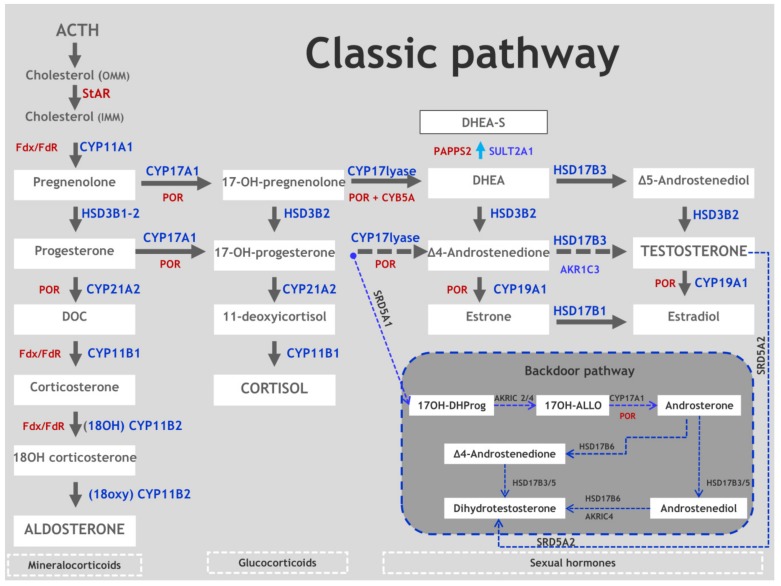
Adrenal and gonadal steroidogenesis (Classic and Backdoor pathways). StAR, steroidogenic acute regulatory protein; OMM, outer microsomal membrane; IMM, inner microsomal membrane; DHEA/DHEA-S, Dehydroepiandrosterone/D-Sulfate; DOC, Deoxycorticosterone; 17OH-DHProg, 5α-Pregnan-17α-ol-3,20-dione (pdiol); 17OH-ALLO, 17OH-allopregnanolone; CYP11A1, cholesterol side-chain cleavage enzyme; CYP17A1, 17α-hydroxylase/17,20 lyase; SULT2A1, dehydroepiandrosterone (DHEA) sulfotransferase; POR, P450 oxidoreductase; CYP21A2, 21-hydroxylase; HSD3B2, 3β-hydroxysteroid dehydrogenase; CYP11B1, 11β-hydroxylase; CYP11B2, aldosterone synthase; HSD17B3, 17β-hydroxysteroid dehydrogenase type 3; SRD5A1, 5α-reductase type 1; SRD5A2, 5α-reductase type 2; Fdx/FdR, ferredoxin/ferredoxin reductase; CYB5A, cytochrome b5.

Steroidogenesis is the process by which cholesterol is transformed into biologically active steroid hormones. Adrenals, gonads, placenta, and other steroidogenic tissues are responsible for a number of common processes with some cell-type-specific variations in each gland [5]. Figure 1 shows both the classical and the backdoor steroidogenic enzymatic steps that apply to adrenal and gonadal steroidogenesis. After the Steroidogenic Acute Regulatory (StAR)-protein-mediated transport of cholesterol from the outer to the inner mitochondrial membrane, the first and rate-limiting step in steroidogenesis is its conversion to pregnenolone (Preg) by a single enzyme, P450scc (CYP11A1) (Figure 1). Subsequently, many other enzymes and cofactors mediate the qualitative production of the type of steroid that is to be produced. Steroidogenic enzymes belong to two groups: cytochrome P450 enzymes and hydroxysteroid dehydrogenases. A cytochrome P450 may be either Type 1 (in mitochondria) or Type 2 (in endoplasmic reticulum), and a hydroxysteroid dehydrogenase may fit in either the aldo-keto reductase family or the short-chain dehydrogenase/reductase family. The activities of these enzymes are modulated by post-translational modifications and cofactors, particularly electron-donating redox partners. The elucidation of the precise roles of these enzymes and cofactors has been significantly facilitated by the identification of the genetic bases of rare disorders of steroidogenesis [5]. Some enzymes that are not primarily involved in steroidogenesis may also catalyze extraglandular steroidogenesis, modulating the phenotype that is expected to result from some mutations [6]. Understanding steroidogenesis is important to our comprehension of disorders of sexual differentiation and other processes that are related to human reproduction and fertility, particularly those that involve androgen excess as a consequence of their impairment.

## 3. 3β-Hydroxysteroid Dehydrogenase Type 2 Deficiency (3β-HSD2D)

3β-HSD2D is a very rare form of CAH that affects less than 5% of all CAH patients with an estimated prevalence of 1/1,000,000 at birth [5,7]. In steroidogenic tissues, it is expressed in both the adrenal glands and the gonads [8].

### 3.1. Genetics

**Figure 2:**
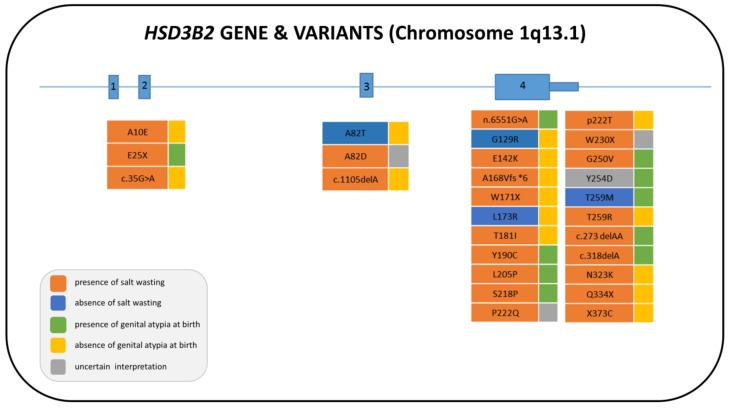
Structure of the human *HSD3B2* gene with the pathogenic variants described in 46,XX. The nomenclature and the color of the boxes of each pathogenic variant are in accordance with the phenotypic characteristics described in each patient, the variant present on the second allele and the references cited in Table S1.

There are two types of human 3β-HSD, which are encoded by two similar (more than 90% sequence identity) genes located on chromosome 1q13.1: *HSD3B1* (encoding 3β-HSD1) is expressed in the placenta and multiple peripheral tissues; and *HSD3B2* (encoding 3β-HSD2) is expressed in the adrenals and gonads. These two enzymes share and catalyze the same reactions, although the Michaelis–Menten constant (*K*m) for 3β-HSD2 is about 10-fold higher than that of 3β-HSD1 [6]. Overall, 43 female patients with or without virilization have been described so far, accounting for 26 reported mutations (Figure 2 and Supplementary Table S1) [9,10,11,12,13,14,15,16,17,18,19,20,21,22,23,24,25,26,27,28,29,30,31,32,33,34,35,36,37]. While severe loss-of-function mutations predict the neonatal salt-wasting (SW) phenotype, for missense mutations the correlation is good only with regard to mineralocorticoid (MC) deficiency.

All reported cases of 3β-HSD deficiency are in the *HSD3B2* [5]; mutations in *3βHSD1* presumably would prevent placental biosynthesis of progesterone, resulting in a spontaneous first-trimester abortion [38].

### 3.2. Biochemistry

In the normal adrenals, the isoenzyme 3β-HSD2 plays a crucial role in all three steroidogenic pathways (aldosterone (Aldo), cortisol (F), and sex hormones production) (Figure 1). It catalyzes two sequential reactions (3β-hydroxysteroid dehydrogenation and Δ5–Δ4-isomerization) of four adrenal Δ5-steroids (pregnenolone (Preg), 17-OHpregnenolone (17-OHpreg), dehydroepiandrosterone (DHEA), and androstenediol (Adiol)), obtaining progesterone (P), 17-α-hydroxyprogesterone (17-OHP), androstenedione (A), and testosterone (T), respectively. The mitochondrial matrix appears to be the preferred location [39,40] for the conversion of Preg to P and the mineralocorticoid production in adrenal steroidogenic cells (Table 1). Interestingly, the *K*m of 3β-HSD2 (5.5 μM) is much higher than that of P450c17 (~0.8 μM), which would favor production of 17-OHpreg and downstream cortisol and sex steroids [6,41]. Similarly, in normal ovarian tissue, cholesterol is converted to Preg, 17-OHpreg, and DHEA. Moreover, 17β-hydroxysteroid dehydrogenase type 3 (HSD17B3)/17β-hydroxysteroid dehydrogenase type 5 (AKR1C3) converts androstendione or androstendiol into testosterone and then, via CYP19A1, into estrogens (Figure 1). Very high levels of Preg, 17-OHpreg, and DHEA are the biochemical markers of 3β-HSD Type II deficiency; however, a high concentration of serum 17-OHP is also paradoxically common in this condition. In fact, some of the secreted 17-OHPreg is then converted to 17-OHP by 3β-HSD1 in peripheral tissues. Plasma levels of A and other Δ4 steroids are also frequently elevated via the same mechanism. However, this elevated serum A may not be effectively taken up by the adrenals and peripheral tissues for the subsequent conversion to T because the circulating concentrations are much less than the Km of AKR1C3, which could explain the lack of virilization in most affected females [42]. Similarly, elevated 17-OHP is not effectively converted to cortisol in the adrenals as the *K*m of P450c21 is low [43]. Urinary steroid profiling, although less used, is similarly accurate and a less-invasive means by which to establish the diagnosis [44].

The principal diagnostic test in 3β-HSD deficiency is intravenous (i.v.) administration of synthetic ACTH with measurement of 17-OHpreg, cortisol, Δ4-17-OHP, DHEA, and androstenedione [7]. Steroidal responses to ACTH cannot reliably identify heterozygous carriers of 3β-HSD deficiency [45].

In general, levels of 17-OHpreg >150 nmol/L, either basal or post ACTH stimulation, is the most reliable single biochemical criterion of true 3β-HSD deficiency [41,46].

### 3.3. Clinical Features and Sex of Rearing

The phenotype of 3β-HSD2D varies, according to the severity of the genetic lesion, from the severe SW form in neonates to mild menstrual disorders in older females [10,11,46,47]. Classically, affected patients present with salt-wasting adrenal crisis, high-renin hypotension, and hypoglycemia, similar to 21-OHD. Affected 46,XX infants may have clitoromegaly and mild virilization because some fetal adrenal DHEA is converted into androstenedione in the periphery via 3β-HSD1, whose expression and function persist in the liver and elsewhere, causing androgen excess [5]. The females, although exhibiting fusion of the labia majora and enlargement of the clitoris, did not have the usual displacement of the urethral orifice as in the urogenital sinus of the other CAH types [47]. With the latter presentation, the diagnosis of 3B-HSD2D is generally done in the first weeks of life. However, this occurrence is not the most common phenotypic manifestation in the neonatal age, as demonstrated by the absence of ambiguous genitalia at birth in most of the cases reported in the literature (Table S1) [42]. Since 3β-HSD2D is a life-threatening condition that could easily be missed in female newborns in the absence of clinical signs of virilization, it is possible that female newborns with some of these severe forms may die undiagnosed due to neonatal adrenal crisis [11]. The low degree of genital atypia of this defect rarely determines sex misassignment (Table S1) and the sex of rearing is female. The low level of fetal hyperandrogenism should not significantly influence gender identity and behavior, although no long-term outcome data are available on this topic for 3β-HSD2D [7].

In the case of the non-SW phenotype, the diagnosis can occur at any pre- or post-pubertal age with symptoms of premature pubarche, hirsutism, and menstrual irregularities, including oligomenorrhea and primary amenorrhea [13]. Since patients with 3β-hydroxysteroid dehydrogenase deficiency may show elevated levels of 17-OHP, it is possible that newborns with false-positive results at neonatal screening for 21-OHD are actually affected by 3β-HSD2D, as confirmed by previous case reports [15,17,48,49].

Non-classical 3β-HSD2D had formerly been hypothesized for young females with premature adrenarche, hirsutism, virilism, or oligomenorrhea based on controversial hormonal criteria showing exaggerated Δ5 steroid production after an ACTH test and elevated 17-OHP/cortisol ratios. However, the *HSD3B1* and *HSD3B2* genes in these patients are normal [8,12,13] and the basis of this condition requires further study [50].

Since HSD3B2 is also necessary for the biosynthesis of sex hormones in the gonads, females may present menstrual irregularities and infertility. There are few long-term follow-up data, which, however, show, in most but not all [13] patients with good therapeutic compliance, a spontaneous process of progressive feminization with menstruation [11,19]. There are no reports of ovarian adrenal rest tumors in 3β-HSD deficiency. Although we know from the literature that HDS3B2D may be associated with obesity, short stature, infertility, or osteoporosis, the rarity of the condition precludes us from establishing its rate of mortality and the cardiovascular health, mental health, and adrenal tumor risk [7].

### 3.4. Therapy and Fertility Prognosis

During the developmental age, the treatment of choice is oral hydrocortisone at slightly higher doses (12–18 mg/m^2^/day) than in patients with 21-OHD; this is probably necessary due to the greater difficulty in suppressing androgens that are generated by the continuous peripheral transformation of DHEA/DHEA-S into testosterone and dihydrotestosterone (DHT) [7]. Long-acting glucocorticoids (prednisone, prednisolone, dexamethasone) might be used in adulthood [11,51], although they maintain the same contraindications that are given for 21-OHD in the most recent guidelines [52]. Administration of fludrocortisone at an average dose of 0.1 mg/day is sufficient to control mineralocorticoid deficiency. In the case of adrenal crisis with loss of salts, rapid rehydration, correction of hypoglycemia, and parenteral administration (IV or IM) of hydrocortisone are necessary. Sex hormone replacement therapy may be necessary in patients with a delay or an absence of pubertal development [13]. It is likely that fertility is reduced, although exact data on this topic are not available [7]. Given the low degree of genital atypia, any cosmetic surgery should be postponed and discussed at the proper time with the person with DSD in the context of a multidisciplinary and experienced DSD team. In the rare cases of severe genital ambiguity, the concerns that will be reported in Section 4.3 on 21-OHD apply.

## 4. 21-Hydroxylase Deficiency (21-OHD)

The most common form of CAH is 21-OHD, which is responsible for about 90% of cases. According to the degree of impairment of 21-hydroxylase activity, three clinical phenotypes are commonly distinguished:

(1) salt-wasting (<1% enzyme activity);
(2) simple virilizing (1–2%); and
(3) non-classical (20–50%).

The classical form (salt wasting and simple virilizing) has a variable incidence, according to ethnicity and location [53,54], and it ranges between 1:20,000 and 1:30,000 female live births [55], with a general heterozygous carriers frequency of 1 in 60 in most populations. The non-classical form generally has a higher incidence, which is estimated to be 1:1000 cases in the Caucasian population (a frequency of heterozygous carriers of 1:10 ) and even greater in other specific ethnic groups (Hebrew Ashkenazi 1:27, Hispanic 1:53, Italians 1:300) [56].

### 4.1. Genetics

The 21-OHD is caused by mutations in the *CYP21A2* gene, which consists of 10 exons and is 3.4 kb long [57,58]. The gene is located on chromosome 6p21.3 within the Class III region of the major histocompatibility complex (MHC) in a complex locus that includes, in addition to the active *CYP21A2* gene, an inactive pseudogene (*CYP21P*) that is about 98% homologous [59,60].

Both genes are arranged in a “tandem repeat” with the *C4B* and *C4A* genes within a repeating module called RCCX. This module consists of one part of or the whole *RP* gene (serine/threonine kinase), an entire *C4* gene, an entire *CYP21* gene, and either one part of or the whole *TNX* gene (tenascin-X).

**Figure 3:**
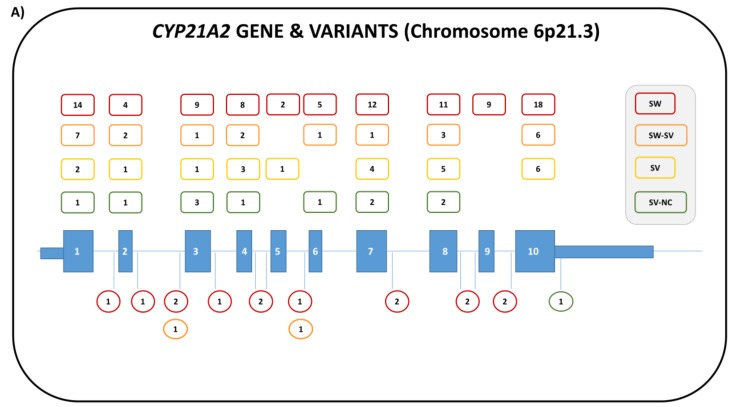

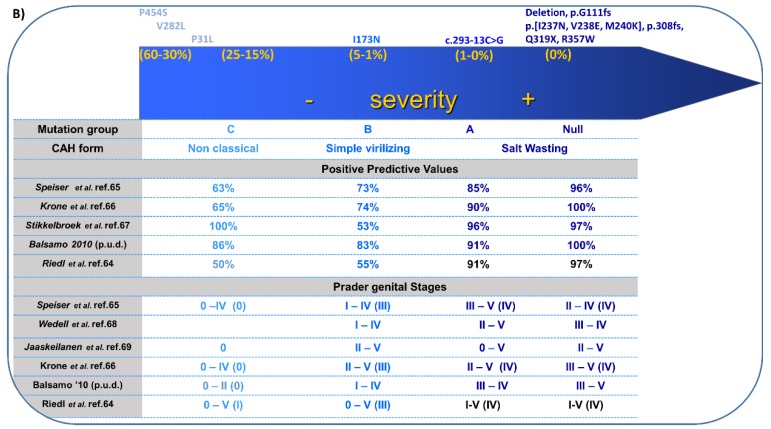
(**A**) Structure of the human *CYP21A2* gene with the number of the pathogenic variants described in both 46,XX and 46,XY patients (modified from Simonetti et al. [59]. (**B**) Genotype/phenotype correlations of *CYP21A2* mutations according to the salt-wasting severity (PPV) in female and male patients and Prader stage classification in 46,XX subjects; p.u.d., personal unpublished data. (modified from Krone and Arlt [66]).

Due to the high homology between the genes and the localization in the MHC region with a high frequency of recombination events, the locus rearrangements, such as gene deletions, duplications, or microconversions from *CYP21P* to *CYP21A2*, are very frequent and account for about 95% of the disease-causing mutations in 21-OHD [2], where about 20% are gene deletions. In addition to the recurrent mutations, another 300 pseudogene-independent rare mutations have been reported to date (Figure 3A) in both sexes [2,59]. Complex alleles, carrying more than one mutation, have also been described. For a correct diagnosis, it is therefore mandatory to analyze the entire *CYP21A2* gene and the allelic segregation of the pathologic variants, and, if necessary, to determine the number of copies of the *CYP1A2* gene. Given the complexity of the locus, in addition, it must be taken into account that, although not all of the rearrangements of the RCCX modules are pathological, they can still significantly interfere with a correct molecular diagnosis. It is, therefore, often necessary to use alternative/additional investigation techniques in order to identify these rearrangements, such as Multiplex Ligation-Dependent Probe Amplification (MLPA) [59,60].

Large deletions and a splicing mutation (c.293-13; A/C > G) are the most common molecular variations associated with the salt-wasting phenotype; loss-of-function mutations that cause the SW phenotype affect heme binding, substrate binding, and membrane anchoring or alter enzyme stability [61,62,63]. A non-conservative amino substitution in exon 4 (p.I73N; 1–2% enzyme activity) is frequently found in patients affected by the simple virilizing form. This phenotype is the result of mutations that affect the transmembrane region or conserved hydrophobic patches and reduce 21-OH activity by 2%. Interference in oxidoreductase interactions, salt-bridge and hydrogen bonding networks, and non-conserved hydrophobic clusters are responsible for the non-classical phenotype, where the enzyme activity is 20–50%. The point mutation in exon 7 (p.V282L) is frequently found in these patients [61,62,63].

Overall, the phenotype is extremely variable [63,64] since the majority (>65%) of CAH patients are compound heterozygotes with one or more mutations in both *CYP21A2* alleles [64,65,66,67,68,69]. In these cases, the clinical phenotype depends on the less severely affected allele [53] (Figure 3B). Variants are reported according to the National Center for Biotechnology Information (NCBI) Reference Sequence NM_000500.9 [59].

In some CAH patients, a particular type of recombination occurs, caused by an unequal crossing-over event between *TNXA* and *TNXB*, that not only eliminates the interposed *CYP21A2* gene, but produces a non-functional chimeric gene *TNXA/TNXB*. Therefore, in this situation, not only is the entire *CYP21A2* gene missing, but a non-functioning *TNX* gene is formed that causes a “contiguous gene syndrome” characterized by CAH and Ehlers–Danlos Syndrome (EDS). This clinically distinct variant of CAH has recently been defined as “CAH-X”. The clinical findings observed in CAH-X patients include joint hypermobility, chronic joint pain, multiple joint dislocations, and structural cardiac valve abnormalities [70]. While homozygous mutations of *TNXB* cause a severe, autosomal recessive form of EDS [71], heterozygosity for *TNXB* mutations causing haploinsufficiency of TNX may be associated with the mild “hypermobility type” of EDS [72]. The CAH-X chimeras cause EDS in an autosomal-dominant manner regardless of CAH status, although patients with CAH usually have more severe EDS manifestations than do carriers without CAH [73,74]. CAH-X variants were initially found in 7–8.5% of patients with CAH [73,74], but this figure has recently been reported to be around 15.6% [75].

Three types of *TNXA/TNXB* chimeric gene have been reported. The first, which was described in 1997 [76] and later termed CAH-X CH-1, has *TNXB* exons 35–44 replaced with *TNXA.* Subsequently, novel *TNXA/TNXB* chimeras, named CAH-X CH-2 and CAH-X CH-3, were identified [77]: CAH-X CH-2 has *TNXB* exons 40–44 replaced with *TNXA,* and CAH-X CH-3 has *TNXB* exons 41–44 substituted by *TNXA* and has been reported in only one patient, with clinical significance still under investigation [70]. CAH-X CH-2 causes a more severe phenotype than CAH-X CH-1 that is characterized by a greater involvement of skin and joints [74].

The use of the commercial MLPA kit (P-050 MRC-Holland) to define *CYP21A2* locus rearrangements is particularly helpful for the identification of a significant portion of CAH-X patients, in particular those that carry the CH-1 chimeric gene. Thus, MLPA may help the clinician provide a diagnosis in early infancy or perform a deep evaluation of clinical signs in adults. An experimental method for detecting both CH-1 and CH-2 has also been recently described [75].

### 4.2. Biochemistry

Both mineralocorticoid (MC) synthesis and glucocorticoid (GC) synthesis depend on CYP21A2 enzyme activity. In the adrenal zona glomerulosa, HSD3B2 converts Preg into P. Then, CYP21A2 converts P to deoxycorticosterone (DOC), which is converted by CYP11B2, which has 11-hydroxylase, 18-hydroxylase, and 18-oxidase activities, into aldosterone (Aldo) [5]. In the adrenal zona fasciculata, Preg is 17-hydroxylated to 17-OHPreg by CYP17A1, which also converts P to 17-OHP. 17-OHPreg is converted into 17-OHP by HSD3B2; CYP21A2 converts 17-OHP into 11-DF, and CYP11B1 finally transforms 11-DF into cortisol [5]. Therefore, in severe 21-OHD, GC and MC would not be sufficiently produced and the increased levels of ACTH, via negative cortisol feedback, would continue to stimulate the adrenal cortex. The excess 17-OHP, which represents the biochemical hallmark of the disease, is converted by the available accessible pathways into potent androgens, such as testosterone (T) and 5α-dihydrotestosterone (DHT). The mechanism by which hyperandrogenism occurs relies on the ready conversion by CYP17A1 of accumulated 17OH-Preg to DHEA (via the Δ5 pathway). Subsequently, DHEA is converted to A by HSD3B2. A very small amount of 17-OHP is converted to A (Δ4 pathway); therefore, this reaction does not represent a significant pathway of androgen synthesis. Both DHEA and A are considered to be weak androgens and a substrate for active androgen synthesis to T by 17β-HSD and, subsequently, DHT by 5alpha reductase type 2 (SRD5A2) in genital skin [78].

An alternative pathway to androgens—‘the backdoor pathway’ (Figure 1)—is responsible for the conversion of 17-OHP to DHT and bypasses the usual intermediate steroids DHEA, A, and T [79]. In this backdoor pathway, 17-OHP is converted to 5α-pregnane-3α, 17α-diol-20-one (pdiol), which is an excellent substrate for the 17-OHP, and 20 lyase activity of CYP17A1 to produce androsterone. After 17β-reduction to 5αAdiol, circulating 5αAdiol is 3a-oxidized to produce DHT, although it is not completely confirmed whether the final conversion of 5alpha-Diol to DHT occurs in genital skin or elsewhere or both [80]. After 17β-reduction to 5αAdiol, circulating 5αAdiol is 3a-oxidized to produce DHT in target tissues such as genital skin and prostate. This alternative pathway to androgens is active in fetal life in healthy individuals and plays a fundamental role in male sex development [79,80]. Androgen production by the backdoor pathway may explain why newborn girls with 21- and 11-hydroxlase deficiencies can be severely virilized, whereas those with 3β-HSD2 deficiency, whose adrenals cannot make 17-OHP, are minimally virilized [13].

In 2012, Kamrath et al. [81] showed that this pathway plays an important role in human hyperandrogenic disorders such as congenital adrenal hyperplasia caused by steroid 21-hydroxylase deficiency. They found significantly elevated ratios of pdiol to the Δ4 and Δ5 pathway metabolites as well as a higher androsterone to etiocholanolone ratio in untreated patients with 21-OHD. The authors found that the elevated pdiol level persisted after one year of age, whereas the androsterone to etiocholanolone ratio was highest only in the neonatal period and in the first year of life. Therefore, they concluded that the backdoor pathway in patients with 21-OHD is primarily activated during infancy.

The interaction between the fetal and the permanent adrenal plays a crucial role in the virilization of genitalia of 21-OHD 46,XX fetuses through both the front-door and the backdoor androgen pathway. In these fetuses, due to the activity of HSD3B2 that is present in the permanent adrenal, DHEA is converted to A [82], which is subsequently converted to T by AKR1C3 (HSD17B5) that is present in the fetal adrenal (the front-door pathway) [83]. At the same time, the high 17-OH progesterone levels activate, primarily in the fetal adrenal, the backdoor pathway to increase the expression of 5αAdiol, which, finally, is 3a-oxidized to produce DHT [79,81].

The utilization of liquid chromatography-tandem mass spectrometry (LC-MS/MS) has allowed us, in recent years, to extensively and more deeply evaluate androgen biosynthesis and the important role in hyperandrogenic disorders of another class of active adrenal androgens: the 11-oxygenated C19 steroids. These androgens derive from the 11-hydroxylation by CYP11B1 of A and T to 11OH-androstenedione (11-OHA4) and 11-hydroxytestosterone (11-OHT), respectively. Subsequently, they are converted by HSD11B2 to 11-ketoandrostenedione (11-KA4) and 11-ketotestosterone (11-KT), respectively. The 11-KT is a potent agonist of the human androgen receptor NR3C4, with an affinity that is comparable to that of testosterone [84,85], and its role as the dominant bioactive androgen in normal and premature adrenarche has recently been reported [86].

**Figure 4:**
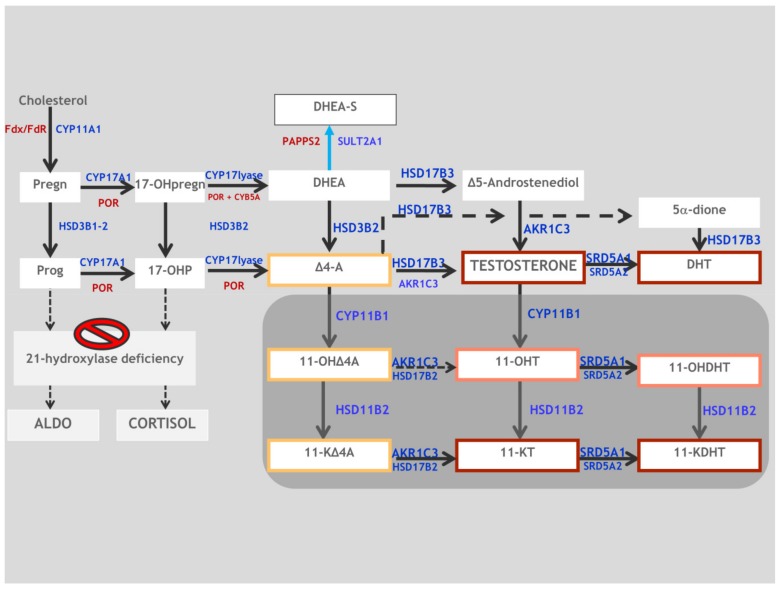
The metabolic pathway of classic and non-classic androgens. The grey box indicates 11-oxygenated C19 steroids. The red, orange, and yellow boxes depict steroids with strong, mild, and weak androgenic activities, respectively. HSD17B3, 17β-hydroxysteroid dehydrogenase type 3; HSD3B2, 3β-hydroxysteroid dehydrogenase type 2; AKR1C3, aldo-keto reductase family 1 member C3; CYP11B1, cytochrome P450 11B1; HSD11B1, 11β-hydroxysteroid dehydrogenase type 1; HSD11B2, 11β-hydroxysteroid dehydrogenase type 2; HSD17B2, 17β-hydroxysteroid dehydrogenase type 2; SRD5A1, 5α-reductase type 1; SRD5A2, 5α-reductase type 2; Pregn, pregnenolone; 17-OHpregn, 17-OHpregnenolone; DHEA-S, dehydroepiandrosterone-sulfate; SULT2A1, sulfotransferase family 2A member 1; DHEA, dehydroepiandrosterone; Prog, progesterone; 17-OHP, 17-OH-progesterone; Δ4-A, Androstenedione; 11-OHΔ4A, 11β-hydroxyandrostenedione; 11-KΔ4A, 11-ketoandrostenedione; 11-OHT, 11β-hydroxytestosterone; 11-KT, 11-ketotestosterone; 5α-dione, 5α-androstanedione; DHT, dihydrotestosterone; 11-OHDHT, 11β-hydroxydihydrotestosterone; 11-KDHT, 11-ketodihydrotestosterone.

In 2016, Turcu et al. demonstrated that adrenal-derived 11-oxygenated 19-carbon steroids are the dominant androgens in classical 21-OHD (Figure 4). In particular, they showed that patients with 21-OHD have higher levels of 11-OHT, 11-KT, 11-OHA4, and 11-KA4 compared to healthy controls, and that 11-KT and 11-OHT only are potent activators of the human androgen receptor [87]. In the last few years, researchers have highlighted the importance and the potential clinical role of 11-oxygenated 19-carbon steroids as more specific markers of adrenal androgen excess compared with DHEA, A, and T, which, at present, are commonly utilized to monitor therapy control in 21-OHD patients [88,89].

Due to the reduced cortisol concentration in the adrenal medulla, CAH patients have impaired activity of medullary phenylethanolamine N-methyltransferase, which catalyzes the conversion of norepinephrine to epinephrine [90]; reduced production of epinephrine may increase the risk of hypoglycemia in these patients during adrenal crisis [90]. The 17-OHP levels directly correlate to the severity of disease, and most classical 21-OHD patients show basal 17-OHP levels above 10,000 ng/dL; basal levels above 200 ng/dL are found in most NC CAH patients, and stimulated 17-OHP levels >1000 ng/dL are useful for confirming the diagnosis [52].

#### 4.2.1. Neonatal Screening

CAH due to 21-OHD can be detected in newborn screening (NS) programs by measuring the amount of 17-OHP, which is now performed in many countries to prevent SW crises during the neonatal period, particularly in males that do not show genital ambiguity, to prevent male sex assignment in affected females, and to reduce long-term morbidities. First-tier screening measures 17-OHP by various techniques, including radioimmunoassays (RIAs), enzyme-linked immunosorbent assays (ELISA), and time-resolved fluoroimmunoassays (DELFIA®), on dried blood spots, and mildly lower levels have been reported in female patients [91]. Diagnosis of 21-OHD can be made when 17-OHP levels are above the cut-off levels that have been elaborated specifically by each screening Centre. Since false-positive results are possible and frequent in premature and severely ill newborns, weight- and gestational-age-adjusted cutoffs for 17-OHP are usually utilized [92,93]. Second-tier strategies that use LC-MS/MS steroid profiling on the same spot sample have recently been developed to improve the positive predictive value of the test [94,95].

#### 4.2.2. Prenatal Diagnosis and Treatment of 21-OHD

The prenatal diagnosis of affected CAH fetuses is possible at 10–12 weeks of gestation by chorionic villous sampling or at 15–16 weeks of gestation by amniocentesis. When, for the first time, cell-free fetal DNA in maternal plasma was detected in 1997, a noninvasive approach to prenatal diagnosis was conceivable [96]. In 2014, Lo et al. developed a noninvasive method for early prenatal diagnosis of fetuses at risk for CAH. With this method, it is possible to determine the inheritance of *CYP21A2* pathogenic variants by noninvasive prenatal testing (NIPT) involving cell-free DNA from maternal blood. The test is performed with a correlative method that utilizes massively parallel sequencing (MPS) of cell-free DNA plasma drawn from an expectant mother. By the same complex technology, it is possible to identify a Y-sequence in maternal plasma via a traditional polymerase chain reaction using an SRY probe. Diagnosis can be made in a few hours and as early as at 5 weeks of gestation [97]. This approach is promising but currently is expensive and feasible only in highly specialized centers. Furthermore, in some countries, prenatal sex determination has been made illegal in order to prevent female feticide [98]. Another approach is preimplantation genetic testing (PGT), which allows for the selection of only embryos that are not affected by CAH for transfer [99].

The prenatal treatment of potentially affected CAH patients aims to prevent virilization of external genitalia of 21-OHD female fetuses by suppressing fetal ACTH and adrenal hyperandrogenism [2]. The treatment protocol involves the administration of a glucocorticoid that is not catabolized by the placenta (dexamethasone) to the mother as soon as pregnancy is suspected, because virilization of genitalia starts from the sixth week of gestation; treatment will be stopped if genetic testing reveals a male or unaffected female fetus [100,101]. In recent years, some important arguments have been raised against prenatal dexamethasone treatment. On the one hand, it reduces or prevents virilization in >85% of treated females [102]; on the other, it can produce negative effects on treated fetuses and their mothers. In line with these findings, in 2012, prenatal dexamethasone treatment in at-risk women was discontinued in Sweden [103]. Subsequent studies have shown that verbal working memory could be affected in children, particularly in girls, without CAH who were treated during the first trimester of fetal life [104,105]. Women with CAH who were treated during the entire gestational period were found to have worse cognitive performances than women with CAH who were not treated prenatally [106]. Furthermore, prenatal dexamethasone treatment in the first three months of life may affect fetal epigenetic programming [107]. Therefore, as stated by the Endocrine Society’s CAH Practice Guidelines [52], the prenatal diagnosis of fetuses who are potentially affected with CAH and their consequent prenatal treatment as formerly carried out in many countries remain controversial. All medical societies that have taken a position on prenatal treatment of 21-OHD with dexamethasone regard it as an experimental therapy that must only be done under research protocols approved by appropriate human experimentation and ethics boards. 

### 4.3. Clinical Features and Sex of Rearing

The clinical picture is often complex and in some cases life threatening. In very severe and very mild cases of 21-OHD, genotype is predictive of phenotype [65]. The intermediate forms are not highly correlated with genotype, suggesting that other factors (environmental or genetic) may contribute to the clinical expression of the disease [2]. The cardinal feature that differentiates patients with classical CAH and patients with non-classical CAH is the genital ambiguity in female newborns with classical CAH. The classical form of 21-OHD is the phenotypic expression of an extremely reduced residual enzymatic activity (REA) of CYP21A2 that is less than 5% of the norm. In salt-wasting CAH, REA is 1–2%. In 46,XX newborns, exposure to an excess of intrauterine androgen causes different degrees of virilization and genital ambiguity as defined by the Prader Scale. Patients with 46,XX CAH have a normal vagina, cervix, and uterus, normal ovaries, and commonly a urogenital sinus and an enlarged clitoris. Secondary to impaired mineralocorticoid production and sodium loss, potassium retention occurs usually within the first 2–4 weeks of life. This metabolic impairment, which is associated with reduced production of epinephrine, can cause hypotension, cardiovascular collapse, and death if left untreated. The diagnosis of CAH due to 21-OHD should be rapidly ruled out in all 46,XX newborns with different degrees of virilization of the genitalia at birth without palpable gonads [52].

In its so-called simple virilizing forms, REA is greater than 2% and less than 5% due to missense mutations, typically p.I173N. Only cortisol deficiency and hyperandrogenism characterize the clinical features of these patients, because production of aldosterone is only moderately impaired [108]. However, for this reason, they could show increased plasma renin activity in the case of a moderate salt restriction. Those 46,XX newborns who are affected by the simple virilizing form show different degrees of virilization of genitalia at birth; 46,XY children affected by the simple virilizing form, which generally goes unrecognized at birth in the absence of a screening program, may be affected by premature pubarche/axillarche or precocious pseudo-puberty with increased growth velocity and rapid maturation of bone age.

The non-classical or late-onset forms show a 21-OH residual activity of >5% and the clinical picture is characterized by hyperandrogenism that is generally recognized in childhood with precocious pubarche/axillarche and advanced bone age. However, in some cases, some degree of external genitalia virilization (clitoral hypertrophy in females) has been described [109].

Regardless of the degree of genital virilization, 46,XX 21-OHD patients are usually raised as females. Since 46,XX 21 OHD patients have a female’s internal anatomy and fertility may be possible, it is still considered inappropriate to classify them as DSD [110]. New guidelines on CAH suggest that clinicians inform and psychologically support parents to help them to cope with the diagnosis and make decisions regarding surgical management of their children [52]. In patients with severe masculinization who are assigned to the female gender, it is generally recommended that surgical correction of internal genitalia be performed early [52] in order to bring the normal vagina to the perineum, repair the fistula between the vagina and the common urogenital channel, and locate the urethra in its normal position [111,112,113,114]. It is important to explain to the patient’s family that, in surgical procedures, the function of genitalia takes priority over their external appearance. Surgical management of clitoridomegaly, even performed by new nerve-sparing procedures, may not be fully satisfactory in terms of sensation, and some groups of CAH patients advocate for postponing this kind of cosmetic surgery until later in life, when the patients can make their own informed decisions. Overall, when these patients are assigned the female sex before 2 years of age and correctly managed by endocrinologists, surgeons, and psychologists who are expert in DSD management, they will generally achieve the female gender identity (88.7%), heterosexual behavior, and a positive psychosocial outcome [115,116]. However, the development of gender identity can only be predicted and not defined during infancy, and some CAH Groups have reported that up to 25% of individuals with CAH do not identify themselves as female [117]. Regarding sexual activities, more than 50% of cases complain of uncomfortable intercourse due to vaginal stenosis (27%) and impairment in the sensitivity of the clitoris. Low pregnancy rates (22.4%) are reported as well [116].

### 4.4. Therapy and Fertility

The goal of CAH treatment is to prevent adrenal crisis and virilization and allow for normal growth and development during childhood to the greatest possible extent.

The administration of glucocorticoids, besides being a substitute for cortisol ‘s own functions, also prevents the progression of virilization in its classical forms from inhibiting androgen hyperproduction by reducing ACTH secretion. However, to achieve this result, maintenance dosages are required that exceed the physiological secretion level of cortisol. Of the various types of cortisones and dosage schemes that have been proposed, the most advisable ones during the growth period consist in three administrations of oral hydrocortisone at doses of 10–15 mg/m^2^/day. Children who are affected by the classical form of 21-OHD also require mineralocorticoid. Newborns and infants require sodium chloride replacement due to MC resistance and immature renal tubules with a reduced sodium reabsorption capacity. Oral fluorohydrocortisone treatment is generally divided into two administrations: from 0.05 to 0.3 mg/day in the neonatal period and from 0.05 to 0.1 mg/day in infancy. Sodium chloride is divided into several administrations in the neonatal period and infancy (1–2 g/day is equal to 17–34 mEq/day).

In patients without an evident electrolyte imbalance, but with supraphysiologic levels of plasma renin activity (PRA), the administration of fluorohydrocortisone is recommended; this group of patients may also include subjects with the “non-classical” form characterized by the Pro30Leu mutation. Monitoring of renin activity, electrolyte levels, and blood pressure can help to evaluate the adequacy of mineralocorticoid replacement therapy.

However, attempts to completely normalize 17-OHP levels frequently result in overtreatment. Patients are at risk of overtreatment and developing Cushingoid side effects, and several evaluations (every 3–4 months in infancy and childhood) of clinical and biochemical features, including 17-OHP, androstenedione, and testosterone, with height velocity and annual bone age assessments after 2 years of age are required to reduce the potential for treatment failure or overtreatment and avoid possible detrimental effects on growth and metabolic health [52].

The reproductive outcome in women with classical CAH has been extensively studied, although conclusive data are lacking on this issue [118]. Fertility is reduced, particularly in patients with the SW form, as a result of several factors, such as hormonal, surgical, psychological, and sexual factors. Menstrual irregularities and anovulation affect from 30% to 75% of women with CAH. Chronically high androgen levels and progesterone overproduction are probably responsible for disturbing the reproductive axis in CAH females. Optimized and individualized glucocorticoid and mineralocorticoid regimens in these patients are needed to allow for spontaneous conception with a successful outcome. Reproductive techniques are now available for subfertile women despite effective adrenal androgen suppression.

The consequences of genital surgery are another factor that is implicated in the reduced fertility of CAH women. Surgery can include clitoroplasty and vaginoplasty, which are often performed in infancy and early childhood. Urinary incontinence, vaginal stenosis and inadequate introitus, poor cosmetics, anorgasmia, and painful intercourse have been reported. In the past few decades, techniques for feminizing surgery have evolved significantly (preserved innervation and clitoral sensation, improved vaginoplasty techniques). In a cohort study of 138 CAH women, Arlt et al. [119] reported that 46% of the participants stated that they were unhappy about their sexual life. In a more recent review of 151 patients with genitoplasty, assessments of cosmetic results showed that the majority of patients (between 60% and 94%) reported good or excellent outcomes [120].

Recently, a large population-based epidemiological study on psychosocial outcomes in CAH patients was conducted in Sweden. A total of 588 CAH patients, including 253 women, were compared to the general population. CAH women, particularly women with the SW form, were less often married, had fewer partnerships, and were less likely to have biological children than controls [121].

In conclusion, higher fertility and fecundity in CAH women will be largely dependent on surgical advances in genital reconstruction, earlier treatment, optimized therapy, the availability of psychological support, and better organization of the transition from pediatric to adult specialist care.

#### Experimental Therapy

To reduce the required doses of glucocorticoids and mineralocorticoids, several experimental treatments have been proposed in conjunction with classical therapies, such as the antiandrogen flutamide and the aromatase inhibitor testolactone [122,123]. In recent years, an extended-release formulation of hydrocortisone, which may help with treatment compliance and reduce the need for more potent glucocorticoids, has been made available [124]. However, Phase 2 and Phase 3 studies are ongoing to evaluate the efficacy and safety of this treatment in 21-OHD adult patients. To help treat younger patients and avoid an improper compounding effect of hydrocortisone from tablets, a very low dose of hydrocortisone in granules with high palatability has been recently released onto the market [125].

## 5. 11β-Hydroxylase Deficiency (11-OHD)

11-OHD, which is caused by mutations in the *CYP11B1* gene, is the second most common cause of CAH with 46,XX DSD in the world, and accounts for about 5% of CAH patients with a European ancestry [126] and for about 15% of CAH patients in the Muslim and Jewish Middle Eastern populations [127]. 17-OHD and StAR deficiency (lipoid CAH) are the second most common form of CAH in China/Brazil and in Japan/Korea, respectively [6]; neither of these deficiencies, however, is causative for 46,XX DSD.

### 5.1. Genetics

**Figure 5:**
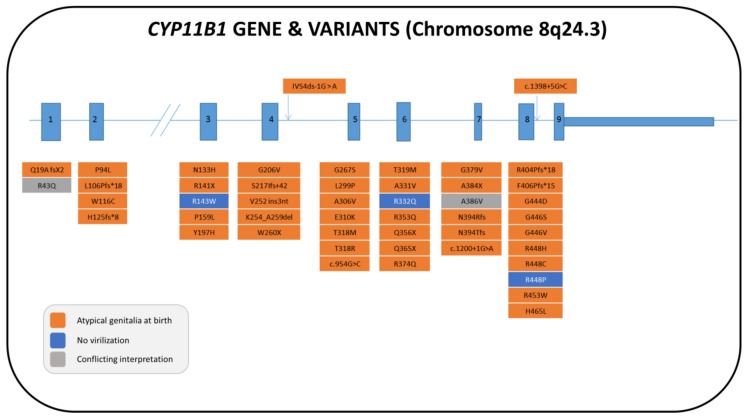
Structure of the human *CYP11B1* gene with the pathogenic variants that have been described in 46,XX patients. The nomenclature of the pathogenic variations is given according to the references cited in Table S2. The nomenclature and the color of the boxes of each pathogenic variant are in accordance with the phenotypic characteristics described in each patient, the variant present on the second allele and the references cited in Table S2.

The *CYP11B1* gene is located on the long arm of chromosome 8 (8q21–q22), and it is in very close proximity to the highly homologous *CYP11B2* gene, which encodes for aldosterone synthase. About 130 mutations of the *CYP11B1* gene have been described and are localized all over the gene, with a minor presence in exons 1 and 9 [128,129,130,131,132,133,134,135,136,137,138,139,140,141,142,143,144,145,146,147,148,149,150]. Figure 5 summarizes the majority of allelic variants that have been described so far in the literature for females (62 patients) together with their influence on genital phenotype (see also Table S2).

A recent review [147] reported that the R448H mutation and eight other mutations (Q356X, G379V, T318M, c.53_54 insT, R454C, R448P, and R148X) are the most frequently found and account for approximately 40% of all cases. *CYP11B1* mutations show significant ethnic specificity, and an updated description of their characteristics can be found in the studies of Khattab et al. [146] and Wang et al. [147]. Recombination events between the two homologous genes have been reported where the *CYP11B1* gene is under the control of the *CYP11B2* promoter such that it responds to angiotensin II and not to ACTH, resulting clinically in classical 11-OHD [151,152].

### 5.2. Biochemistry

In the normal adrenals, 11β-Hydroxylase is expressed in the zona fasciculata and converts 11-deoxycortisol to cortisol in response to ACTH (Figure 1). Aldosterone synthase is expressed at low levels in the zona glomerulosa and catalyzes the 11-hydroxylation, 18-hydroxylation, and 18-methyl oxidase activity needed to convert DOC to Aldo in response to angiotensin II and potassium. Both 11β-hydroxylase and aldosterone synthase can convert DOC into corticosterone (B). 11β-hydroxylase also has weak 18-hydroxylase activity; however, only aldosterone synthase can synthesize Aldo from 18OH-corticosterone [6]. 11-OHD disrupts the synthesis of cortisol with normal production of Aldo. ACTH excess due to a cortisol deficit causes overproduction of androgens and DOC, which causes virilization in females and hypertension, respectively. 11-deoxycortisol, which is hyper-responsive to ACTH administration, is the key steroid used in diagnosis [6]. Elevated serum B, DOC, and 17-OHP are also present, and it is possible for a wrong diagnosis of 21-OHD to occur with newborn screening for an accumulation of 17-OHP. Useful for diagnosis are urinary metabolites, such as tetrahydrocortisone, tetrahydro-11-deoxycorticosterone, and tetrahydro-11-deoxycortisol [3].

### 5.3. Clinical Features and Sex of Rearing

The classical form of 11-OHD has an estimated frequency of 1:200,000 live births [142] and is characterized by androgen excess and hypertension. Androgen excess causes ambiguous genitalia at birth in affected females (46,XX DSD) [3] and precocious pseudo-puberty with rapid somatic growth and bone age acceleration. Hypertension might not be apparent during the neonatal period due to mineralocorticoid resistance, and some patients can present with salt loss during the neonatal period [153]. Usually, sex assignment in these patients is female in accordance with the advice given for 21-OHD (Section 4.3 and Table 2).

The non-classical form of 11-OHD is similar to the non-classical form of 21-OHD with signs of androgen excess, including mild virilization, hirsutism, precocious pseudo-puberty, menstrual irregularities, and polycystic ovary syndrome in affected females. In this mild form, arterial hypertension does not occur often [154,155].

### 5.4. Therapy and Fertility Prognosis

Treatment of 11-OHD is glucocorticoid replacement at doses similar to those used in 21-OHD (orally administrated hydrocortisone at doses of 10–25 mg/m^2^/day) to ameliorate hypothalamic–pituitary–adrenal axis feedback to control androgen excess and hypertension; however, mineralocorticoid replacement is not needed [6]. Sometimes, treatment with mineralocorticoid receptor antagonists may be necessary [129]. As for 21-OHD, genital surgery may be necessary, but the timing of the surgery remains controversial. New guidelines for CAH suggest that clinicians inform parents about surgical options, including delaying surgery and/or observation until the child is older [52]. The existing studies underline that a significant number of individuals with CAH and the female gender identity evaluate their early surgery as a positive. However the development of gender identity can only be predicted and not defined during infancy; 25% of individuals with CAH do not identify themselves as female [117]. If the diagnosis of 11-OHD is late, it is possible that patients with a high Prader score will be reared as boys. There are some case reports in which these patients continued to be boys after diagnosis, and so the uterus and ovaries were removed [156].

Data on fertility are scarce, and only one successful pregnancy has been reported in the literature [157]. Caution should be taken when spironolactone is used to manage high blood pressure due to its teratogenic potential.

## 6. Aromatase Deficiency (AroD)

AroD is a rare genetic condition that is caused by mutations in the *CYP19A1* gene, which is located on chromosome 15q21.1. Aromatase enzyme converts androgens into estrogens in many tissues. AroD causes atypical genitalia in 46,XX fetuses and maternal virilization during pregnancy due to an increased concentration of androgens. Ovaries are usually large and polycystic in girls with AroD [158].

### 6.1. Genetics

**Figure 6:**
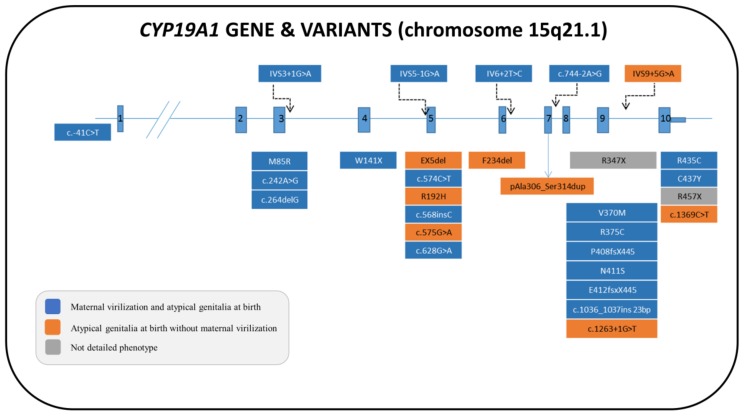
Structure of the human *CYP19A1* gene with the pathogenic variants that have been described in 46,XX patients. The nomenclature and the color of the boxes of each pathogenic variant are in accordance with the phenotypic characteristics described in each patient, the variant present on the second allele and the references cited in Table S3.

Aromatase deficiency is an autosomal recessive disorder that was first described by Shozu et al. [159]. To date, 30 46,XX cases with various ethnic origins have been described, and more than 29 distinct pathogenic variations have been identified in the *CYP19A1* gene, including missense, nonsense, small deletions and insertions, splice-site mutations, and one large intragenic deletion [158,159,160,161,162,163,164,165,166,167,168,169,170,171,172,173,174,175,176,177,178,179,180]. Most of these mutations have been found to be located in exon 5 and exon 9 [173] (Figure 6 and Table S3).

### 6.2. Biochemistry

Aromatase catalyzes the three precursors androstenedione, testosterone, and 16-α-hydroxy dehydroepiandrosterone sulfate (after conversion to 16-α-hydroxyandrostenedione) into estrone, estradiol, and estriol, respectively [158,181]. Fetuses that lack aromatase activity are not able to convert the DHEA-S that is produced by the fetal adrenal gland into estrogens in the placenta; DHEA-S is, therefore, converted to testosterone, resulting in the virilization of both the fetus and the mother.

Both basal and GnRH-stimulated FSH levels have been shown to be higher in girls with AroD during the first two years of life compared to normal subjects (50–75 and 200–255 mIU/mL, respectively). However, the estradiol and estrone levels tend to be remarkably low during this same period [161,167]. Moreover, basal LH is often within normal limits or slightly elevated during infancy (5–10 mIU/mL). A previous study showed that, in a girl with AroD, the FSH and LH levels persistently increased and multicystic ovaries developed between the ages of three and four years [161]. However, Belgorosky et al. and Lin et al. [167,169] reported that the basal FSH and LH levels in a girl with AroD were found to be increased during mini-puberty and to show a dramatic decrease between two and five months of age. In the Unal et al. report [179], gonadotropin (FSH, LH) levels were found to be elevated since birth. The persistence, in some described cases, of high levels of gonadotropins, in particular of FSH, through infancy and childhood could suggest that a prolonged effect of an androgen or a lack of estrogen might result in an irreversible influence on the GnRH pulse generator. Moreover, an amplification of FSH signaling might occur in the presence of high intraovarian androgen production, and this mechanism could be involved in the development of ovarian follicular cysts [158].

### 6.3. Clinical Features and Sex of Rearing

The clinical characteristics of patients with aromatase deficiency vary depending on gender, age, and enzymatic activity [158]. AroD leads to an increase in intrauterine androgen concentration, which results in varying degrees of postnatal virilization in the external genitalia in girls. During infancy and childhood, there are usually no symptoms of aromatase deficiency, although some girl patients may present with abdominal symptoms of ovarian cysts because of mild changes in the hypothalamic–pituitary–gonadal axis due to a lack of feedback regulation [182]. AroD may lead to a number of clinical conditions in adolescent girls, such as delayed puberty, hypergonadotropic hypogonadism, multicystic ovaries, and primary amenorrhea in accordance with estrogen deficiency. Signs of virilization, such as acne, hirsutism, and cliteromegaly, in accordance with androgen excess, may also be present [158,181]. Estrogen deficiency, on the other hand, causes delayed epiphyseal closure, eunuchoid body habitus, osteopenia, and osteoporosis that develop in both genders [165].

In most fetuses with AroD, early-onset (12 weeks) or late-onset (up to 30 weeks) maternal virilization may be noted [161]. The non-aromatized fetoplacental and maternal androgen precursors are converted to testosterone in the placenta and also in peripheral maternal tissues, resulting in maternal virilization. After giving birth, the signs of virilization disappear gradually and the androgen levels return to normal [158].

There are few data in the literature on the possible effects of foetal hyperandrogenism in the programming of the brain for sexual identity and behavior. Although no detailed psychosexual studies have been reported, female psychosexual orientation has been observed and the female sex of rearing has been realized in most 46,XX subjects. Only one female patient, described by Lin et al. [169], with the homozygous F234del mutation, was raised as a male; this was probably due to the severe masculinization of the external genitalia at birth (Prader stage IV). This patient reported male gender identity, role, and orientation but experienced significant breast development (Tanner stage IV) at puberty that required a mastectomy, a salpingo-oophorectomy, and a hysterectomy. Tritiated androstenedione assays showed that this F234del mutant had between 16% and 19% wild-type activity, consistent with the significant uterine growth and breast development seen in this case. However, the same authors were not able to establish whether, in choosing the sex of rearing, the objective effect of prenatal androgen exposure coupled with estrogen insufficiency or social and cultural influences were predominant.

### 6.4. Therapy and Fertility Prognosis

To date, information on the effect of estrogen administration to prevent the consequences of estrogen insufficiency is limited and ambiguous. There is no consensus on the appropriate dosage of estrogen replacement therapy and on the usefulness of starting low-dose estrogen treatment from infancy and childhood in affected patients. Therapeutic follow-up data are available in 10 reported female patients who had been treated with estrogen at different ages in prepuberty and puberty [176]. In most cases, the treatment was performed in premenarcheal girls aged from 9.6 to 14 years to induce puberty. Generally, increasing doses of oral-conjugated estrogen have been used according to clinical signs of estrogen response, and progestogens were added to induce menarche. This therapeutic model, however, proved to be inadequate in about half of the cases to improve bone maturation delay, suppress elevated gonadotropin levels, regress ovarian cysts, and induce menarche. In the Janner et al. study [182], a girl with complete AroD started low-dose estrogen therapy at the age of 3.5 years and continued the treatment until the age of 15 years with increasing doses. The authors suggested that a low dose of estrogen (E2) (50–100 mg/day), even if insufficient to suppress gonadotropin feedback, is required for normal longitudinal growth and bone age maturation during early childhood, while higher E2 doses (1.4–2 mg/day) are needed more for successful negative feedback on the pituitary–ovarian axis, ovarian cyst regression, breast development, and endometrial reflex than for the normalization of growth and the appearance of menarche in late prepuberty and puberty.

Information about the course of the disease in adulthood and the long-term consequence for fertility remain unknown. However, the clinical, hormonal, and therapeutic problems that are observed in most patients, along with the surgical consequences of genital reconstruction, might affect reproductive capacity in these women [158].

## 7. P450 Oxidoreductase Deficiency (PORD)

In humans, PORD causes an unusual and rare form of CAH, whose exact incidence is unknown [3]. Its presentation is characterized by DSD in both sexes and is often associated with skeletal defects [183,184].

### 7.1. Genetics

**Figure 7:**
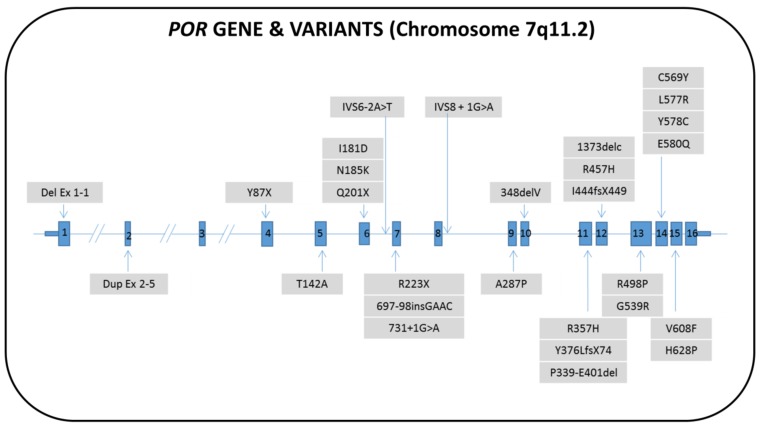
Structure of the human *POR* gene with the pathogenic variants that have been described in 46,XX patients. The nomenclature of the pathogenic variants is in accordance with the references cited in Supplementary Table S4. The genotype/phenotype correlation is not shown, due to the high variability of the reported cases.

The genetic cause of this new form of CAH was discovered in 2004 [183,185] and explained the pathogenesis of a condition that, so far [186], has been referred to as “apparent associated 17α- and 21-hydroxylase deficiency”. The *POR* gene is located on the long arm of chromosome 7 (7q11.2). About 80 female patients affected by PORD with more than 30 different pathogenic variants have been reported to date (Figure 7 and Table S4) [183,184,185,187,188,189,190,191,192,193,194,195,196,197,198,199,200,201,202,203,204,205,206,207].

The phenotypic result in genetic females characteristically depends on the causative *POR* pathogenic variant [208]. In fact, depending on the type of mutation and its location within the *POR* gene, it may reduce the CYP17A1, CYP21A2, and CYP19A1 activities differently. Therefore, *POR* variants must be studied separately for each potential P450 target enzyme [204]. Interestingly, the *POR* mutation p.A287P, which characterizes European patients [183,188], alters CYP17A1 activity but not CYP21A2 activity or CYP19A1 activity as opposed to the *POR* mutation p.R457H, which is frequent in Japanese patients [184,189] and instead predominantly reduces CYP19A1 activity [209]. The latter process allows for an increase in androgens in the maternal circulation and virilizes the mother. Therefore, pregnant women with fetuses carrying the *POR* p.R457H mutation frequently show virilization during pregnancy [183,184,185,189]. Furthermore, the deviation of steroidogenesis towards the “backdoor pathway” (Figure 1) contributes to the virilization of mothers and affected female fetuses [189]. The ethnic variability and the high polymorphism of the *POR* gene was confirmed by the high recurrence of p.A503V sequence variation in mildly affected patients of Chinese origin, but not in those of African origin, as also occurs for incidences of other SNPs [6]. As for the “skeletal” phenotype, the greater the severity of the involved mutations, greater the severity of the “skeletal” phenotype [188,210]. Severe phenotypes can lead to stillbirth or early neonatal deaths [211]. Prenatal testing is possible through molecular genetic techniques once the *POR* pathogenic variant has been established for a known family [188].

### 7.2. Biochemistry

POR is a protein that has flavin adenine dinucleotide (FAD) and flavin mononucleotide (FMN) portions; the FAD transfers two electrons received from NADPH to the FMN, which, in turn, transfers them to all microsomal (type 2) CYP enzymes [212,213], including the steroidogenic enzymes CYP21A2, CYP17A1, and CYP19A1.

Measurements of serum and urinary steroid profiles have shown that POR deficiency is characterized by partially deficient CYP17A1 activity, with or without associated deficiencies of CYP21A2 and CYP19A1 enzymes [183,184,185,188]. POR-deficient patients typically have a normal mineralocorticoid function, low–normal cortisol levels that respond weakly to an ACTH stimulation test, high concentrations of 17-OHP that respond unpredictably to ACTH, and low levels of C19 precursors of sex steroids (Table S4). Anomalous elevations in some of the steroids (and their metabolites) from the "backdoor pathway" may support a diagnosis of POR deficiency [190]. The low estriol values seen in women carrying a fetus with some *POR* mutations may highlight a CYP19A1 deficiency and hyperandrogenism during pregnancy. Hyperandrogenism is explained by the production of 5α-reduced androgens via the alternative pathway in the fetus that cannot be aromatized by the placenta and is transferred to the maternal circulation [183,184,185,189].

Urinary steroid profiling by gas chromatography-mass spectrometry has disclosed the characteristic trait of a combined impairment of CYP21A2 activity and CYP17A1 activity [44,188] and a typical accumulation of pregnenolone metabolites (pregnanediol, PD) [44].

Prenatal testing is possible using maternal urinary steroid profiling from 12 weeks onwards [211]. Neonatal screening for 21-OHD may occasionally detect 17-OHP values above the cut-off level in patients with PORD [183,193].

Although POR is required by the main hepatic drug-metabolizing enzymes and several in vitro studies of these enzymes have shown a major impairment by *POR* mutations [208,213,214], effects in PORD patients are rare [215].

### 7.3. Clinical Features and Sex of Rearing

46,XX babies are born virilized without postnatal progression of virilization. This result depends on the existence of the alternative “backdoor pathway” (secondary to CYP21A2 deficiency), which leads to DHT production and is active only during the prenatal period [185,216]. After birth, the pathway’s activity declines; thus, a sex steroid deficiency appears in both sexes. During puberty, females can present with delayed development of sexual characteristics, often showing significant hypergonadotropic hypogonadism and presenting large ovarian cysts with a tendency to torsion [187,192]. In addition, pregnant mothers carrying a fetus affected by PORD may manifest virilization during pregnancy (see Section 7.2), which characteristically resolves postpartum [183,185,187,210]. Although hyperandrogenism declines after birth, the genital phenotype can range from Prader stage I to V. The majority of reported cases show Prader stages below IV and a female sex assignment. In a few cases, patients that scored Prader IV/V were assigned the male sex and subsequently shifted to the female sex after multidisciplinary and parental counselling (Table S4). No long-term outcome is available at present to determine the influence of prenatal androgenic imprinting on the gender identity of these cases.

Newborns with PORD often have an associated skeletal defect that has been described as part of the Antley–Bixler Syndrome (ABS) phenotype. Craniofacial anomalies involve brachycephaly, proptosis, choanal stenosis, and craniosynostosis with midface hypoplasia, which most commonly involves the coronal and lambdoid sutures and can be complicated by the hydrocephalus in severe forms [210]. Other frequent skeletal abnormalities may involve large joint synostosis, such as radio-humeral synostosis, congenital bowing of the femurs, hand and foot malformations such as long palms, camptodactyly, and arachnodactyly, and rocker bottom feet. A scoring system for classifying the severity of the Antley–Bixler syndrome phenotype has been proposed based on the number and severity of abnormal skeletal features [188].

ABS is a skeletal condition that can occur in two forms: the first is transmitted as an autosomal recessive trait [183,187,216], is associated with atypical genitalia in both sexes due to defects in steroidogenesis, and is caused by mutations in the *POR* gene; the second is transmitted as an autosomal dominant trait, does not involve steroidogenesis, and is caused by gain-of-function mutations in the fibroblast growth factor receptor 2 *(FGFR2*) gene [184,187].

PORD impairs CYP enzymes involved in sterol synthesis, causing retinoic acid accumulation at embryonic locations that usually form skeletal joints and sutures, leading to their premature fusion and the ABS skeletal phenotype [210,217]. In addition, PORD affects a number of key CYP enzymes involved in hepatic function (CYP1A2, CYP2C9, CYP2C19, CYP2D6, and CYP3A4) that metabolize a large number of the xenobiotics and drugs that are used in clinical practice [218].

### 7.4. Therapy and Fertility Prognosis

Treatment of PORD requires a multidisciplinary team of endocrinologists, geneticists, and orthopedic and cranio-facial surgeons and family support. The majority of affected patients (about 90%) will require low-dose glucocorticoid replacement therapy; 50% of them will need permanent, and the other 50% at least some, stress-related hydrocortisone replacement therapy; this should be determined individually through the assessment of cortisol response to ACTH, regardless of presence or absence of DSD [214]. Females may require estrogen replacement during puberty, preferably as an estrogen patch to avoid hepatic first-pass metabolism. This is important from the therapeutic point of view; in fact, since CYP3A4 metabolizes estrogens and glucocorticoids (GCs), it is likely that individuals with *POR* mutations affecting this enzyme activity may have a reduced hepatic clearance of these hormones and, consequently, higher than expected circulating levels [215].

Atypical genitalia may require surgical intervention, which should be discussed with the patient and the patient’s parents in the context of an experienced multidisciplinary team. Also, for this condition, information about the clinical course of the disease in adulthood and the long-term consequence for fertility remain unknown and the clinical, hormonal, and therapeutic problems that are observed in most patients, along with the surgical consequences of genital reconstruction, might affect reproductive capacity in these women.

Skeletal abnormalities may require physical and specialized therapy or surgical intervention.

## 8. Conclusions

46,XX DSD secondary to androgen excess represents a group of congenital conditions with atypical sex development. In these cases, an early diagnosis is mandatory not only for an appropriate sex assignment, but also to start the correct substitutive treatment to avoid life-threatening adrenal crisis. In most of these monogenic conditions, hormonal phenotypes correlate quite well with genotypes. Molecular analysis certainly is fundamental to a confirmation of the diagnostic suspicion and should firstly rely on an appropriate biochemical investigation. Patients with 46,XX DSD should be referred to a specialized Center where appropriate (i.e., tandem mass steroid profiles) treatment can be performed and skilled pediatric endocrinologists and geneticists lead a multidisciplinary team that can definitively manage these children from diagnosis to adulthood.

## Figures and Tables

**Table 1:** Disorders of steroid metabolism that cause androgen excess (Modified from [4]).

Group/Name of IEM	Enzyme	Gene/Inheritance	Cellular/Tissue Localization	OMIM Gene Number
3β-hydroxysteroid dehydrogenase 2 deficiency (3β-HSD2D)	3βHSD2 or HSD3B2	*HSD3B2/*AR	Mitochondria/each adrenal zone and gonads	613,890
21-hydroxylase deficiency (21-OHD)	P450c21 or CYP21A2	*CYP21A2/*AR	Endoplasmic reticulum/each adrenal zone	613,815
11β-hydroxylase deficiency (11-OHD)	P450c11β or CYP11B1	*CYP11B1/*AR	Mitochondria/adrenal fasciculata	610,613
Aromatase deficiency (AroD)	P450aro or CYP19A1	*CYP19A1/*AR	Endoplasmic reticulum/gonads (granulosa cells)	107,910
Cytochrome P450 oxidoreductase deficiency (PORD)	P450-OR or POR	*POR/*AR	Endoplasmic reticulum/each adrenal zone and gonads	124,015

**Table 2:** A summary of clinical and biochemical features, therapy, and clinical course of the disease in 46,XX differences of sex development (DSD).

Enzymatic Deficiency	Clinical Deatures at Birth	Other Vlinical Features	Biochemical Markers for Diagnosis	Clinical Course	Therapy	Sex of Rearing	Fertility
**3β-HSD2D**	Salt wasting (70%); mild genital atipicity (25%)	Precocious adrenarche;	*Serum*: ↑ stimulated ratio of Δ4 over Δ5 steroids; *Urine*: ↑ ratios DHEA/GC metabolites and 5PT/GC metabolites	Hirsutism, polycystic ovaries, primary amenorrhea, or irregular menses; osteopenia/osteoporosis	Hydrocortisone Fludrocortisone +/− Rapid rehydration, correction of hypoglycemia +/− Sex hormone	female	Unknown
**21-OHD**	**Classical**: salt wasting + atypical genitalia (75%); simple virilization (25%). **Non-classical**: asymptomatic	Enlarged adrenals; pseudo-precocious puberty; adrenal rest in the ovaries	*Serum:* ↑17-OHP and 21-DOF; 11-KΔ4A, 11-KT *Urine:* ↑ metabolites of 17-OHP (17HP,PT) and 21-DOF (P’TONE) *Saliva*: ↑11-KΔ4A and 11-KT	Precocious puberty; hirsutism; irregular menses; polycystic ovaries	Hydrocortisone Fludrocortisone +/− Rapid rehydration, correction of hypoglycemia	Prader 1–3: female. Prader 4–5: early diagnosis tentatively female; late diagnosis: possible male	Generally reduced according to the severity of enzymatic deficiency; improvement with therapeutic optimization and compliance
**11-OHD**	**Classical**: atypical genitalia (100%), high blood pressure (~10%) **Non-classical**: asymptomatic	Pseudo-precocious puberty; high blood pressure in infancy (20%)	*Serum:* ↑ S and DOC *Urine:* ↑ metabolites of S (THS) and DOC (THDOC)	Precocious puberty; hirsutism; irregular menses; polycystic ovaries; high blood pressure (50%)	Hydrocortisone +/− Mineralocorticoid receptor antagonists	As above	As above; caution with spironolattone due to teratogenic effects
**AroD**	Various degrees of external genitalia atipicity (100%)	Gestational maternal virilization; abdominal signs of ovarian cysts	*Serum*: ↑ T; +/− ↑ LH and FSH	Delayed puberty; primary amenorrhea; osteopenia/osteoporosis; dislypidemic pattern	Increasing doses of estrogens; progestins added to induce menache	female	Unknown
**PORD**	Various degrees of external genitalia atipicity (~75%)	Gestational maternal virilization; skeletal malformations of the Antley–Bixler phenotype	*Serum:* unspecific mild ↑ of 17-OHP; ↑ Pregn, Prog, 17-OHP *Urine:* combined impairment of diagnostic ratios for CYP17A1D and CYP21A2D; ↑ of Pregn metabolites (PD)	Declining androgenization after birth; hypergonadotropic hypogonadism with delayed puberty; large ovarian cysts	+/− Hydrocortisone +/− Sex hormone	female (declining virilization after birth); concerns about prenatal androgenic imprinting	Unknown

GC, glucocorticoid; Δ4 steroids: progesterone, 17-OHP, and androstenedione; Δ5 steroids: pregnenolone, 17-OHpregnenolone, and DHEA; 5-PT, 5-pregnentriol; 21-DOF, 21-deoxycortisol; 11-KΔ4A, 11-ketoandrostenedione; 11-KT, 11-ketotestosterone; 17HP, 17OH.pregnanolone; PT, pregnanetriolone; P’TONE, pregnanetriolone; S, 11-deoxycortisol; DOC, 11-deoxicorticosterone; THS, tetrahydro-11-deoxycortisol; THDOC, tetrahydrocorticosterone; PD, pregnanediol.

## Supplementary Materials

Supplementary materials can be found at https://www.mdpi.com/1422-0067/20/18/4605/s1.

## Graphic Abstract Illustration Credits

From Servier Medical Art (https://smart.servier.com/), reproduced under Creative Commons License attribution 3.0 Unported License.
