# Supplementary material for: 46,XX DSD Due to Androgen Excess in Monogenic Disorders of Steroidogenesis: Genetic, Biochemical, and Clinical Features
Source: Int J Mol Sci. 2019 Sep 17;20(18):4605. doi: 10.3390/ijms20184605 (PMC6769793; doi:10.3390/ijms20184605)
Supplement: Supplementary file 1 [file ijms-20-04605-s001.pdf]

Table S1. Types of *HSD3B2* pathogenic variants, biochemical and clinical parameters in female patients reported in the literature.

Case n.	Genotype/variants	Age at presentation	Phenotype		Hormone levels			Reference
			Adrenal	DSD	17Prog/ Δ4A/T/17Preg/DHEA/DHEA-S (nmol/L)	ACTH / F (pmol/L-nmol/L)	Renin / Na / K (pmol/L-mEq/L)	
1	A10E/A10E	3 weeks<br>4 months	SW	Normal genitalia	B: 302.6/ nd / nd / 772.8 /nd / nd<br>B: 127.3 / nd / 2.9 / nd / nd / 13,400	99.0 / 772.8<br>96.4 / 227.8	nd / 122 / 9.0<br>nd /129 / 6.3	[11]
2	E25X/E25X	Neonatal period	SW	Clitoromegaly	NA	NA	NA	[14]
3-12	c.35G>A/c.35G>A	The majority detected by CAH NS	SW	Normal genitalia; postnatal PCOS/ hirsutism	NA	NA	NA	[15]
13	A82T/A82T	31 years	NSW	Normal genitalia	NA	NA	NA	[16]
14	A82T/A82T	5 years	NSW	Premature Pubarche (PP)	NA	NA	NA	[16]
15	A82D/W230X	14 days CAH NS	SW	Normal genitalia §	B: 760 / 53 / >55 /640.6 / 5.8 / nd	180 / 400	600 / 127 / 6.0	[17]
16	G129R/P222Q	7 years	NSW	Normal genitalia;<br>PP at 7 years	B: 3.0/ 1.7 / 0.4 / 69 /19.1 / 0.8<br>S: 2.1 / 3.5 / nd / 339 / 30.2 / nd	B: nd / 300<br>S: - / 500	NA	[41]
17″	G129R/P222Q	6.7 years	NSW	Normal genitalia;<br>PP at 6.7 years	B: 8.5 / 1.4 / 1.3 / 90 / 18 / 500<br>S: 23.0 / 1.7 / nd / 351 / 29 / nd	B: nd / 500<br>S: - /700	NA	[41]
18	G129R/ n.6551G>A	8 years	NSW	Normal genitalia;<br>PP at 4 years	B: 4.6 / 4.9 / nd / 103 / 108.4 / nd<br>S: 12.8 / 6.3 / nd / 294 / 115.0 / nd	NA	NA	[12,46]

19	E142K/ Stop373C	CAH NS	NSW	Normal genitalia;<br>PP at 5 years	B: 19.1/ 1.75 / nd /180/ 29.3/ nd<br>S: 35.1 / 2.3 / nd / 760 / 72.4 / nd	B: nd / 390<br>S: - / 750	NA	[21,46]
20	W171X/W171X	13 days	SW	Normal genitalia<br>hyperpigmentation	NA	NA	NA	[10]
21″	W171X/W171X	24 days	SW	Normal female<br>hyperpigmentation	NA	NA	NA	[10]
22	W171X/ A168Vfs *6	8 days – CAH NS	SW	No genital virilization	B: 124 /nd / nd / nd / nd/ 12,000	549ng/L / 92	116pg/mL/136/5.8	[18–20]
23	L173R/L173R	2 years	NSW	Normal female	NA	NA	Na	[22]
24	T181I/c.1105delA	7.6 years	SW	PP; growth accel.;<br>No genital virilization	B: 27.3 / 4.4 / 1.5 / 275.1 / 37.0 / 6898<br>S: 24.6 / 4.0 / nd / 235.8 / 23.1 / nd	B: 46 /373<br>S: nd / 293	317	[23]
25″	T181I/c.1105delA	3.6 years	SW	No genital virilization	B: 34.7 / 3.8 / 0.8 / 210.4 / 11.9 / 5825<br>S: 30.9 / 3.6 / nd / 141.3 / 13.7 / nd	B: 81 / 551<br>S: nd / 421	1750	[23]
26	Y190C/S218P	CAH NS	SW	Clitoromegaly;<br>labial fusion	B: 66.6 / 3.91 / nd / 910 / 263/ nd	32.5/193.0	34.4*/139/5.1	[24]
27	L205P/L205P	11 months	SW	Hyperpigmentation;<br>mild clitoromegaly	B: elevated 17preg /17OHP ratio	Elevated ACTH	NA	[25]
28	P222Q/P222Q	1 month<br>7 months	SW	Mild clitoromegaly;<br>first diagnosis 21-hydroxylase deficiency	B: 5.6/ nd / nd / 195.2 / nd / nd	NA	NA	[13]
29	P222T/P222T	4 weeks	SW	No genital virilization	B: 184 / 48 / nd / 1102 /385.5/ nd	NA	NA	[21,46,48]

30	P222T/P222T	CAH NS	SW	No genital virilization	181/-/-/292			[21,46,48]
31	G250V/G250V	7 months	SW	Postnatal clitoromegaly, precocious pubarche	B: 427.2 / 40.8 / 12 / nd / nd / 143,000	635.4/132.5	10.05/135/5	[26]
32	Y254D/ not found	Puberty	NSW	Primary amenorrhea, Mild clitoromegaly and hirsutism, enlarged polycystic ovaries	B: 91.3 / 17.1 / nd / 968.5 / 270.7/ 32.8	nd / 228.9	NA	[27–29]
33	T259M/T259M	41 years	NSW	Clitoromegaly, severe virilization	B: nd / 70.2 / 23.0 / 234.4 / 901.0 / 55,900	B: 137 / nd		[30]
34	T259M/T259M	7.8 years	NSW	PP at 3 years, mild clitoromegaly	B: 3.6 / 3.8 / 1.6 /153 / 35/ 4.6<br>S: 24.5/ 4.2 / nd / 147 / 38/ nd	B: nd /100<br>S: - / 100	NA	[31,32,41]
35	T259R/T259R	2 weeks	SW	Normal genitalia with severe pigmentation	NA	NA	NA	[13,33]
36	c.273 delAA/c.318delA	10 days	SW	Mild clitoromegaly, PP at 7 months	8 years (Rx)<br>B: <0.15 / <0.18 / <0.17 / 0.39 /1.37/ <140<br>S: <0.15 / <0.18 / <0.17 / 1.38 / 1.65 / <140	B: nd / 110<br>S: nd / 80	NA	[34]
37	N323K/N323K	11 days	SW	No genital virilization	B: 784 / >41 / 1.07/ nd / nd / nd	NA	/128/6.8	[35]

38″	N323K/N323K	7 days	SW	No genital virilization	B: 987 / nd / 0.58 / nd / 2,562 / nd	197 / nd	/ 129 / 5.7	[35]
39	Q334X/Q334X	13 days<br>CAH NS	SW	Normal female	B: 185.8°-82.3/ 84.1 / nd / nd / 0.55 / nd<br>S: DHEA 2.53	nd / nd	27.4*/135/6.8	[36]
40″	Q334X/Q334X	28 days<br>1 year	SW	Normal female	B: nd / nd/ 5.1 / nd / nd / nd	nd / 25.3	nd/ 109 / 8,94	[36]
41	Q334X/ Q334X	25 days	SW	Normal female	B: DHEAS >100	NA	nd / 135 / 7.7	[37]
42	Q334X/ Q334X	CAH NS<br>8 years	SW	Normal female; focal segmental glomerulonephritis	NA	NA	NA	[37]
43	Q334X/ R335X	2 months	SW	Normal female	B: nd / nd / nd / 31.3 / nd / nd	78.3 / nd	nd / 120 / 5.9	[37]

″: sister of the case reported in the above line, respectively; SW: salt wasting; DSD: genital phenotype; CAH NS: neonatal screening for CAH; 17Prog: 17OH-progesterone; Δ4A: androstenedione; T: Testosterone; 17Preg: 17OH-Pregnenolone; DHEA/-S: dehydroepiandrosterone/-sulphate; F: cortisol; B: basal level; S: post 60′ ACTH test (250 μg iv) level; * Plasma Renin Activity (PRA): μg/L/h; ° paper disc assay; nd: not done; Rx: treated with low dose Hydrocortisone; §virilization after therapy withdrawal.

Table S2. Types of *CYP11B1* pathogenic variants, biochemical and clinical parameters in 46,XX patients reported in the literature.

Case n. (#)	Genotype/variants	Age at presentation (yrs.) /Country of origin	Phenotype			Steroid hormone levels		Ref.
			age at BP measure (yrs.)	SBP/DBP	DSD (Prader)	DOC (nmol/L)	S (nmol/L)	
1 ARG1A	R43Q; A386V; R453W/ CYP11B2-CYP11B1 chimera	0/Caucasian	0.25	98/56	Atypical genitalia (4)	ND	433	[146]
2 ARG1B	R43Q; A386V; R453W/ CYP11B2-CYP11B1 chimera	0/Caucasian	0.333	100/75	Atypical genitalia (4)	ND	86.3 [1]	[146]
3 BRA1	Q356X /Q356X	1/Brasil	36	160/110	Atypical genitalia (ND)	ND	909	[146]
4 BRA2	G267S/G267S	ND/Brasil	6	100/60	Atypical genitalia M‡ (5)	ND	54.8	[146]
5 BRA3	R404Pfs*18/R404Pfs*18	0/Lebanon	11	140/110	Atypical genitalia (3)	ND	333.7	[135]
6 CAN3	c.1200+1G>A/c.1200+1G>	2.5/Arabic Pakistani	2.5	118/57	Virilization (2)	52	964	[146]
7 EGY1	R347Q/R347Q	0 /Caucasian/Egypt	3	70/50	Atypical genitalia M‡ (4)	ND	520	[146]
8 EGY2	G446V/G446V	0 /Caucasian/Egyptian	1	94/70	Atypical genitalia (4)	ND	517	[146]
9 EGY3	G446V/G446V	0.08/Caucasian/Egyptian	0.5	90/60	Atypical genitalia (4)	ND	78	[146]

10 EGY4	G446V/G446V	0.08/Caucasian/Egyptian	1	90/50	Atypical genitalia (3)	ND	78	[146]
11 EGY6	R448H/R448H	0/Caucasian/Egyptian	1	75/55	Atypical genitalia M‡ (4)	ND	465	[146]
12 EGY8	R448H/R448H	3/Caucasian/Egyptian	ND	ND	Atypical genitalia M‡ (5)	N/A	548	[146]
13 EGY9	N133H/T319M	0.16/Caucasian/Egyptian	0.333	83/53	Atypical genitalia (4)	ND	468	[146]
14 IRN1	R43Q/A386V	0/Persian, Iranian	0.75	170/90	ND (4)	ND	ND	[146]
15 IRN4	R448C/R448C	1.83/Persian, Iranian	2.16	160/100	ND (4)	ND	ND	[146]
16 IRN8	V252ins3nt/V252ins3nt 46,XX (83%)/45,XO (17%)	0/Persian, Iranian	ND	ND	Atypical genitalia M‡ (5)	ND	ND	[146]
17 IRN31	W116C/W116C	0/Persian, Iranian	12.42	170/110	Atypical genitalia (4)	ND	ND	[146]
18 SAU1 A	H465L/H465L	0/Saudi	0.5	105/78	Atypical genitalia (3)	ND	107	[146]
19 SAU2 A	Q19Afs*21/Q19Afs*21	14/Saudi	11	126/69	46,XX M (5) Family screen as his sister was diagnosed	ND	520	[140]
20 SAU2 B	Q19Afs*21/Q19Afs*21	10/Saudi	2.66	95/48	46,XX M (5) Family screen as his sister was diagnosed	ND	476	[140]

20 SAU2 C	Q19Afs*21/Q19Afs*21	16/Saudi	16	140/88	46,XX M‡ (4) Breast Tanner IV, short stature, hypertension	ND	219	[140]
21 SAU4 A	R448P/R448P	7/Saudi	9	103/69	Clitoromegaly, diagnosed at birth with 21OHD (1)	ND	37	[146]
22 SAU4 B	R448P/R448P	0/Saudi	0.069	97/74	Clitoromegaly (1)	ND	670	[146]
23 SAU6	W260X/W260X	1.33/Saudi	0.5833	98/65	Atypical genitalia (4)	ND	621	[146]
24 SAU7	G206V/G206V	1.08/Saudi	1.25	106/46	Atypical genitalia (3) 46,XX sex ND	ND	214	[146]
25 TUN1	S217Ifs*42/S217Ifs*	0/Arab-Berber/Tunisian	2	120/90	Atypical genitalia (3), melanodermia 46,XX sex ND	ND	387	[138]
26 TUN2 A	G379V/G379V	0/Arab-Berber/Tunisian	2	120/60	Atypical genitalia (ND), hyperpigmentation; 46,XX sex ND;	ND	ND	[146]
27 TUN2 B	G379V/G379V	0/Arab-Berber/Tunisian	6	140/100	Atypical genitalia (ND), 46,XX sex ND	ND	ND	[146]
28 TUN3 A	G379V/G379V	0/ Arab-Berber/Tunisian	ND	Normotensive	Atypical genitalia (ND), 46,XX sex ND	ND	ND	[146]

29 TUN3B	G379V/G379V	0/ Arab-Berber/Tunisian	ND	ND	Atypical genitalia (ND), hyperpigmentation, virilization 46,XX sex ND	ND	ND	[146]
30 TUN4B	G379V/G379V	1.08 /Arab-Berber/Tunisian	3	135/90	Atypical genitalia (3), precocious puberty, 46,XX sex ND	ND	ND	[146]
31 TUN5A	G379V/G379V	0/Arab-Berber/Tunisian	ND	ND	Atypical genitalia (4) 46,XX sex ND	ND	ND	[146]
32 TUN6	G379V/G379V	0/Arab-Berber/Tunisian	ND	ND	Atypical genitalia (ND), hyperpigmentation, 46,XX sex ND	ND	ND	[146]
33 TUN7	G379V/G379V	0/Arab-Berber/Tunisian	ND	ND	Atypical genitalia (4) 46,XX sex ND	ND	ND	[146]
34 TUN8A	G379V/G379V	0/Arab-Berber/Tunisian	ND	ND	Atypical genitalia (ND) 46,XX sex ND Died at 4 months of age	ND	ND	[146]
35 TUN9A	G379V/G379V	0/Arab-Berber/Tunisian	ND	ND	Atypical genitalia (ND) 46,XX sex ND	ND	ND	[146]
36 TUR1	R141X/R141X	0.41 /Caucasian/Turkish	0.416	90/40	Atypical genitalia (4)	ND	289 a	[146]
37 TUR2	R141X/R141X	4.2/Caucasian/Turkish	4.3	120/80	Accelerated growth, penis enlargement, pubic hair (5); 46,XX M	ND	289	[146]

38 TUR4	L299P/L299P	2.7/Caucasian/Turkish	2.7	160/110	Penis enlargement, pubic hair, nonpalpable gonads (5); 46,XX M	ND	1,045	[146]
39 TUR5 A	L299P/L299P	2.2/Caucasian/Turkish	ND	ND	Atypical genitalia (5) 46,XX M‡	ND	852	[146]
40 TUR8 A	G446S/G446S	4.9/Caucasian/Turkish	4.9	80/60	Premature adrenarche after index case (her brother) (4)	ND	517	[146]
41 USA1 A	A331V/A331V	0/SephardicJewish/Egyp tian	0	76/45	Sibling/atypical genitalia (4) 46,XX M‡	4.4	ND	[146]
42 USA1 B	A331V/A331V	1.16/Sephardic Jewish/Egyptian	ND	ND	Atypical genitalia (3)	267	ND	[146]
43 USA2	A331V/A331V	0/Sephardic Jewish, Turkish/Syrian	ND	ND	Atypical genitalia (4)	2†	170	[146]
44 USA3	A331V/A331V	0/Sephardic Jewish, Syrian	3.42	110/60	Atypical genitalia (3)	31	ND	[146]
45 USA4	A331V/A331V	0/Sephardic Jewish/Egyptian	ND	ND	Atypical genitalia (ND)	4†	ND	[146]
46 USA6 A	T318M/T318M	0/Yemeni	1.02	110/65	Atypical genitalia 4	61	ND	[146]

47 USA6B	T318M/T318M	0/ Yemeni	0	80/50	Sibling/atypical genitalia 4	16	ND	[146]
48 USA6C	T318M/T318M	Prenatal /Yemeni	ND	ND	Sibling (1§)	5†	ND	[146]
49 USA7	L106Pfs*18/T318M	0/German, Dutch/Canadian Dutch	2	101/62	Atypical genitalia (3)	7	ND	[146]
50 USA8A	F406Pfs*15/F406Pfs*15	0/Mexican	0.0055	67/38	Atypical genitalia (ND)	1.1†	ND	[146]
51 USA8B	F406Pfs*15/F406Pfs*15	0/Mexican	0.0055	61/35	Atypical genitalia (ND)	38	1,152	[146]
52 USA9	F406Pfs*15/F406Pfs*15	0/Mexican/Mexican	0.0027	73/44	Atypical genitalia (3) 46,XX M‡	7	58	[146]
53 USA11	Q365X/G444D	0 African American	12	150/90	Atypical genitalia (2)	28	ND	[132]
54	K254_A259del/K254_A259del	N/A/Kwait	11	140/100	Atypical genitalia (5) 46,XX M			[136]
55	P159L/P159L	7/East German	ND	ND	Premature pubarche (1)			[136]
56	IVS4ds-1G >A/IVS4ds-1G >A	5/ Brazil	5	High	Atypical genitalia (2) and precocious puberty,	63	92	[150]

57	Q356X/Q356X	0.7/Brazil	0	normal	Atypical genitalia (3) and salt wasting	ND	72	[150]
58	Y197H/R353Q	23/China	13	180/120	Normal female (1)	ND	ND	[147]
59	R143W/A306V	6/Italy	ND	ND	Normal female (1); acne, accelerated growth, adv. BA	ND	ND	[142]
60	L299P/R332Q	5,5/Italy	5.6	100/65	Normal female (1); premature pubarche	324s	294s	[142]
61	E310K/L299P	0.36/Italy	1	80/50	Atypical genitalia (3)	ND	754†	[142]
62	Q356X/A306V	0.24/Italy	10	110/70	Atypical genitalia (3)	ND	951†	[142]
63	I318R/IVS6-1G>C	0/Italy			Atypical genitalia (4)	766†	165†	UC
64	R384X/Q356X	2.6/Colombia	2,6	>99th pct	Atypical genitalia (5) 46,XX M	High	ND	[144]

(#) The code that follows "case n." refers to the original list of patients included into the corresponding reference. BA, bone age; BP, blood pressure; CA, chronological age; DBP, diastolic blood pressure; DOC, 11-deoxycorticosterone; N/A, not applicable; ND, not determined; S, 11-deoxycortisol (compound S); SBP, systolic blood pressure; SD, standard deviation. UC, unpublished case. †On medication. ‡Reassigned to female after being reared as male. §Prenatally treated.

Table S3. Genotype/Phenotype spectrum in CYP19A1 deficient females who were prepubertal or pubertal at diagnosis *(from Belgorosky A et al,* [158] *modif.).*

Case n.	Genotype/variants	Aromatase activity (%)	Presentation	Phenotype	Pitutary-gonads axis	Ref.
				Mother; patient		
1	IV6+2T>C/IV6+2T>C	<0.3	prepubertal	Maternal virilization; Atypical genitalia at birth;	↑ androgens in cord serum	[159,160]
2	R435C/ C437 Y	1.1/0	pubertal	Maternal virilization (?); Atypical genitalia at birth; Puberty absent; Ovarian cysts and virilizing signs at pubertal age	↑ FSH ↑ androgens in childhood	[161,162]
3	R375C/R375C	0.2	pubertal	Maternal virilization; Atypical genitalia at birth; Puberty absent; Ovarian cysts and virilizing signs at pubertal age	ND	[163]
4	R457X /R347X	-	prepubertal	Not detailed phenotype reported	ND	[161]
5	P408fsx445 /IVS3+1G>A	0	prepubertal	Maternal virilization; Atypical genitalia at birth; Ovarian cysts during childhood	↑ androgens in cord serum<br>↑ FSH ↑ androgens in childhood	[165]
6	V370M/V370M	-	prepubertal	Maternal virilization (?); Atypical genitalia at birth	ND	[166]
7	c.628G>A/E412fsX445	-	prepubertal	Maternal virilization;	↑ LH, FSH ↑ androgens in infancy	[167,168]

				Atypical genitalia at birth; Bone age delay; Spontaneous puberty; Ovarian cysts and virilizing signs at pubertal age; Metabolic syndrome		
8	R435C/R435C	0.7-1.5	pubertal	Maternal virilization; Atypical genitalia at birth; Bone age delay; Spontaneous puberty; Ovarian cysts	↑ FSH ↑ androgens in childhood	[169]
9, 10	F234del/F234del	16-19	a.prepubertal<br>b.pubertal	a.Atypical genitalia at birth<br>b.Atypical genitalia at birth; Bone age delay; Spontaneous puberty; Ovarian cysts	-	[169]
11	EX5del/EX5del	-	pubertal	Atypical genitalia at birth; Bone age delay	Low androgen levels	[169]
12	M85R/IVS5-1G>A	0	prepubertal	Maternal virilization; Atypical genitalia at birth; Bone age delay; Puberty absent; Virilizing signs at pub. age	-	[170]
13	-41 C>T/p.N411S	0<br>50	prepubertal	Maternal virilization; Atypical genitalia at birth; Bone age delay	Normal LH, FSH, androgens in childhood	[171]
14, 15	p.Trp141* /p.Trp141*	0	prepubertal	Maternal virilization; Atypical genitalia at birth	↑ FSH, normal androgens in childhood	[180]

16	R192H/R192H	19	prepubertal	Atypical genitalia at birth	↑ androgens at birth ↑ FSH, normal androgens in childhood	[172]
17	pAla306_Ser314dup (homoz. or hemiz)	-	adult	Atypical genitalia at birth; Puberty absent; Metabolic syndrome	↑ LH, FSH, ↑ androgens	[173]
18, 19	c.628G>A/c.628G>A	-	pubertal	Atypical genitalia at birth; Bone age delay; Spontaneous puberty; Ovarian cysts	↑ LH, FSH, ↑ androgens	[174]
20	c.574C>T/c.574C>T	0	prepubertal	Maternal virilization; Atypical genitalia at birth	↑ FSH, normal androgens in childhood	[174]
21	c.574C>T/c.1369C>T	-	pubertal	Atypical genitalia at birth; Bone age delay; Spontaneous puberty; Ovarian cysts	↑ FSH, normal androgens in childhood	[174]
22	c.628G>A/c.242A>G	-	prepubertal	Maternal virilization; Atypical genitalia at birth; Bone age delay	↑ FSH, normal androgens in childhood	[174]
23	IVS9+5G>A/IVS9+5G>A	-	prepubertal	No maternal virilization; Atypical genitalia at birth; Bone age delay; Spontaneous initial puberty; Ovarian cysts	↑ LH, FSH, early pubertal basal estradiol levels at 13.5 yrs.	[175]
24	c.264delG/23-bp insertion	0	prepubertal	Maternal virilization; Atypical genitalia at birth (Prader 4); bone age delay	↑ FSH, normal androgens in childhood	[176]

25	c.568insC/c.568insC	0	prepubertal	Maternal virilization, death during labor (acute hemolysis);<br>Atypical genitalia at birth (Prader 2); severe cerebral palsy, failure to trive	Normal LH, FSH, normal androgens	[177]
26	c.568insC/c.568insC	0	prepubertal	Maternal virilization;<br>Atypical genitalia at birth (Prader); hypoplastic ovaries	↑ LH, FSH, normal androgens	[177]
27	c.568insC/c.568insC	0	prepubertal	Maternal virilization;<br>Atypical genitalia at birth (Prader 3);	↑ LH, FSH, normal androgens	[177]
28	c.1263+1G>T/c.1263+1G>T	0	adult	Atypical genitalia at birth; grown as a male; streak gonads;	↑ LH, FSH, T at normal female levels	[179]
29	c.744-2A>G/c.744-2A>G	0	prepubertal	Maternal virilization;<br>Atypical genitalia at birth (Prader 4)	↑ FSH ↑ androgens in infancy<br>↑ FSH, normal androgens in childhood	[179]

ND, not determined.

Table S4. Genotypes, plasma steroid hormone profiles and clinical features of reported patients with 46,XX karyotype and PORD.

Case n.	Genotype/variants	Age at presentation (yrs.)/ Country of Origin	Phenotype		Steroid hormone levels (nmol/L)								Ref.
			DSD Pt°/M″	Skeletal Abn..^	F basal	F peak	17OHP basal	17OHP peak	DHEA	DHEAS*	A	T ; E2*	
**01**	731+1G→A/R357H	0./Japan	Y/Y	Y	L (229)	L (287)	H (25)#	*na*	*na*	*na*	*na*	*na*; L	[183]
**04**	C569Y/V608F	23/Brazil	N/*na*	N	N (386)	L (469)	H (17)	H (42)	*na*	*na*	L (0.7)	N (0.9) ; *na*	
**01**	R457H/A287P	16/Poland	Y/N	M	L (290)	L (436)	H (26)	H (53)	*na*	N (5.7)	N (2.6)	*na*; L (53)	[180,182]
**03**	C569Y/I181D	0/Germany	Y/*na*	M	L (291)	L (280)	N (5.0)	*na*	*na*	N	N	N ; *na*	
**16**	A287P/ NF	0./UK	Y/*na*	Y	N (220)	L (534)	H (43)	*na*	*na*	*na*	*na*	*na* ; *na*	[184]
**03**	Y578C/I444fsX449	0.5 /Japan	Y/*na*	Y	N (295)	L (350)	H (33)	H (63)	L (0,7)	*na*	N (1.7)	H (1.7); N (48)	[189]
**06**	R457H/R457H	2.0/ Japan	Y/*na*	M	N (287)	L (397)	H (15)	H (97)	L (<0.3)	*na*	N (1.0)	N (0.3); L (<36)	
**07**	R457H/ NF	6.7/Japan	P/*na*	Y	N (228)	L (196)	H (24)	H (41)	N (3.1)	*na*	L (<0.3)	N (<0.3); L (<36)	
**08**	R457H/R457H	8.9/Japan	Y/*na*	M	N ((231)	L (289)	H (19)	H (122)	*na*	*na*	*na*	N (<0,3); L (<36)	
**09**	R457H/R457H	0.6/Japan	Y/*na*	Y	N (286)	L (375)	H (63)	H (169)	N (6.2)	*na*	H (6.3)	N (1.4); L (<36)	
**10**	R457H/R457H	1.2/Japan	P/*na*	B	H (618)	N (772)	H (34)	*na*	N (3.1)	*na*	H (9.4)	H (1.7); N (205)	
**02**	R457H/R457H	0.3/Japan	Y/Y	M	N (326)	L (536)	H (15)	H (111)	N (2.4)	*na*	H (3.5)	N (1.0); N (80)	[207]
**03**	R457H/R457H	0.3/Japan	Y/Y	M	N (390	L (654)	H (15)	H (108)	N (8.3)	*na*	N (1.8)	N (0.3); N (<40)	

**17**	R457H/Q201X	0.8 (Japan)	P/N	B	Hormonal measurements are not individually available								[190]
**21**	R457H/E580Q	12 years (Japan)	N/N	Y									
**01**	A287P/NF + <u>*CYP21A2* del/P30L+In2s</u>	18 days (Canada)	P/N	M	*na*	*na*	H 1,69)	*na*	*na*	N (0.8)	H (13.8)	N (1.7); *na*	[191]
**11**	R457H/R457H	3.0/Japan	About 50% of Pts. & Ms. showed virilization	M	Hormonal measurements are not individually available; in general, 17-OHP was normal or elevated at the baseline and above the normal range after ACTH stimulation, and cortisol was normal at the baseline but barely responded to ACTH stimulation.								[192]
**12**	R457H/R457H	0.2/Japan		M									
**13**		0.1/Japan		M									
**14**	R457H/R457H	18/Japan		M									
**27**	R457H/R457H	4.2/Japan		Y									
**28**	R457H/ NTrascr.	17/Japan		Y									
**34**	R457H/ I444fsX449	0.7/Japan		Y									
	R357H/348delV												
**02**	N185K/L577R	19/Caucasian	N/*na*	N	L (267)	L (234)	H (36)	H (71)	*na*	*na*	N (2.6)	N (0.8); N (92)	[193]
**03**	G539R/1373delC	17/Ireland	Y/*na*	Y	N (339)	L (391)	*na*	*na*	*na*	N(2.6)	N (4.5)	*na*; N (40)	
**04**	G539R/697-98insGAAC	0/Cauc./Australia	Y/*na*	Y	L (63)	L (430)	N (4.7)	H (20)	*na*	L (<0.8)	*na*	*na; na*	
**01**	P399-E401del/ “	1.5/Turkey	Y/*na*	M	N (331)	L (276)	H (93)	H (85)	N (5.5)	L (0.082)	N (1.0)	H (1.6); *na*	[194]
**02**	P399-E401del/ “	10.5/Turkey	Y/*na*	Y	N(327)	L (300)	H (74)	H (71)	L (0.7)	L (0.23)	N (2.0)	N (<0.3); *na*	
**02**	A287P/A287P	12/Poland	Y/*na*	M	N (607)	N (717)	H (72)	H (87)	*na*	N (2.4)	N (3.4)	*na*; L (<50)	[187]
**03**	A287P/A287P	23/Italy	Y/N	M	L (149)	L (158)	H (68)	H (82)	*na*	L (2.0)	*na*	L (0.5); *na*	
**04**	T142A/Y376LfsX74	19/Netherlands	N/*na*	Y	N (375)	N (425)	H (25)	H (36)	*na*	L (1.2)	L (0.6)	N (1.1); N (90)	
**05**	A287P/R223X	16/Germany	Y/N	Y	L (190)	L (314)	H (15)	*na*	*na*	N (4.2)	N (1.7)	N (0.5 ; N (84)	

**05**	A287P/ NF	0/Germany	Y/Y	Y	*na*	L (127)	H (20)	*na*	*na*	*na*	*na*	*na*	[183,1
**06**	A287P/IVS6 -2 A>T	0/USA	Y/Y	Y	N (462)	L (512)	*na*	*na*	*na*	*na*	*na*	*na*	91,194
**11**	A287P/H628P	0/USA	Y/Y	Y	L (170)	L (209)	H (31)	*na*	*na*	*na*	*na*	*na*	]
**13**	A287P/Del ex 1-1	0/Netherlands	N/*na*	M	N (607)	N (717)	H (72)	*na*	*na*	*na*	*na*	*na*	
**14**	A287P/IVS8 + 1 G>A	0/UK	Y/*na*	Y	*na*	*na*	H (39)	*na*	*na*	*na*	*na*	*na*	
**15**	A287P/A287P	0/USA	Y/*na*	Y	*na*	*na*	*na*	*na*	*na*	*na*	*na*	*na*	
**18**	A287P/A287P	0/USA	Y/*na*	*na*	L (121)	L (303)	*na*	*na*	*na*	*na*	*na*	*na*	
**21**	Y87X/NF	0/Austria	Y/*na*	N	L (149)	L (158)	H (68)	*na*	*na*	*na*	*na*	*na*	
**25**	R498P/R498P	0/Pakistan	Y/*na*	Y	N (495)	L (548)	*na*	*na*	*na*	*na*	*na*	*na*	
**30**	A287P/Dup ex2-5	31/Netherlands	Y/*na*	Y	L (360)	L (350)	H (20)	*na*	*na*	*na*	*na*	*na*	
**02**	R454H/1698insC	11/Japan	Y/na	Y	Hormonal diagnostic workup by urinary steroid analysis								[200]
**01**	R457H/I444fsX449	0.6/Korea	YS/N	Y	L (257)	L (265)	H (68)	H(110)	*na*	L (0.3)	L (0.3)	N (0.3); *na*	[201]
**01**	R457H/R357H	0/Nepal	Y/Y	Y	L (143)	*na*	H (58)	*na*	*na*	*na*	*na*	N (0.3); *na*	[206]
**01**	A287P/R457H	0/Romania	Y/Y	Y	N (nr)	*na*	H (nr)	*na*	*na*	N (nr)	*na*	N (nr); *na*	[202]
**01**	R223X/M408K	0.6/Brazil	YS/Y	B	L (248)	*na*	H (35)	*na*	N (2.1)	*na*	N (0.7)	N (0.07); *na*	[203]
**01**	A287P/IVS7 c.732-2A>T	Prenatal/France	Y/*na*	Y	*na*	*na*	*na*	*na*	*na*	*na*	*na*	*na*	[204]
**01**	A457H/ G177A	21/Korea	N/*na*	Y	N (403)	L (361)	H (16)	*na*	*na*	*na*	*na*	*na*	[205]

This table includes reported female patients for whom there are some published plasma hormone data; the case n. refers to the corresponding paper . "**N**" means the value was in the normal range; "**L**" means the value was below the normal range; "**H**" means the value was above the normal range; "nr" and "*na*" indicate that the hormonal value was not reported or measured, respectively. When available, the measured specific hormone level have been reported in brackets . Stimulated = Value after stimulation with ACTH; F = cortisol (divide by 2.759 to obtain ng/mL); 17OHP = 17OH progesterone (divide by 3.026 to obtain ng/mL); DOC = deoxycorticosterone; DHEA = dehydroepiandrosterone (divide by 3.467 to obtain ng/mL); DHEAS = dehydroepiandrosterone sulfate (divide by 2.714 to obtain µg/mL); A = androstenedione (divide by 3.491 to obtain ng/mL); T = testosterone (divide by 3.467 to obtain ng/mL); E2 = Estradiol (divide by 3.671 to obtain pg/mL). NTrascr.= not transcribed; NF = Not found; *All hormone levels are expressed in nmol/L except for DHEAS (µmol/L) and E2 (pmol/L).

° Pt = Postnatal presence (Y =clitoromegaly + labial fusion; P = partial virilization with clitoromegaly or labial fusion; S: M to F change) or absence (N) of virilization in Patients; “ M = presence of virilization during pregnancy in Mothers (Y). # positive to neonatal CAH screening; ^ Skeletal abnormalities: Y = overt at birth, M = mild, B = borderline.
